# NET-derived DNA is associated with cGAS-STING and type I IFN responses, as supported by monocyte stimulation cultures using plasma from female patients with SLE

**DOI:** 10.1016/j.ebiom.2026.106507

**Published:** 2026-10-07

**Authors:** Xiaolin Cao, Dagmar Duijzer, Tineke Kardol-Hoefnagel, Bettina C. Geertsema-Hoeve, Jeanette H.W. Leusen, Maarten Limper, Marianne Boes

**Affiliations:** a Center for Translational Immunology, University Medical Center Utrecht, Utrecht University, the Netherlands; b Department of Rheumatology and Clinical Immunology, University Medical Center Utrecht, Utrecht University, the Netherlands; c Department of Internal Medicine, Flevo Hospital, Almere, the Netherlands; d Department of Pediatrics, University Medical Center Utrecht, Utrecht University, the Netherlands

**Keywords:** Neutrophil extracellular traps (NETs), cGAS-STING pathway, Monocytes, Type I interferons (IFNs), Systemic lupus erythematosus (SLE), NET-Associated DNA

## Abstract

**Background:**

Neutrophil extracellular traps (NETs) are implicated in type I interferon (IFN) production in Systemic Lupus Erythematosus (SLE). While NETs are known to activate plasmacytoid dendritic cells (pDCs) via binding to Toll-like receptors, their effects on other immune cells remain underexplored. This study investigated whether NETs in plasma from patients with active SLE trigger cGAS-STING signalling in monocytes and induce the production of type I IFN and other immunomodulatory cytokines.

**Methods:**

Monocytes from healthy donors were cultured with diluted plasma from healthy donors or patients with SLE across varying disease activity, or with *in vitro*-generated NETs. Activation of the cGAS-STING pathway was assessed by flow cytometric analysis of phosphorylated STING (pSTING) and TBK1 (pTBK1). Cytokine production, including type I IFNs, was quantified using bead-based immunoassays. Only female donor samples meeting the study eligibility criteria were available for inclusion in this cohort.

**Findings:**

Plasma MPO-DNA levels correlated with IFNβ levels in patients with SLE (P = 0.002). SLE plasma induced STING and TBK1 phosphorylation, and IFNβ expression in healthy monocytes, with responses increasing according to donor disease activity (P < 0.0005). Experiments with healthy monocytes exposed to *in vitro*-generated NETs recapitulated these findings, evidenced by increased pSTING and pTBK1 expression and enhanced IFNα and IFNβ secretion (P < 0.05), which were inhibited by cGAS-IN-1.

**Interpretation:**

Our findings support a role for NET-associated DNA in activating cGAS-STING signalling and promoting type I IFN production by monocytes in SLE. These findings identify NET-mediated cGAS-STING signalling in monocytes as a pathway that warrants further investigation in the context of type I IFN-driven inflammation in SLE.

**Funding:**

Not Applicable.

**Research in context**

**Evidence before this study**

Systemic lupus erythematosus (SLE) is characterised by dysregulated type I interferon (IFN) responses. Previous studies have demonstrated that neutrophil extracellular traps (NETs) are increased in SLE and can contribute to inflammation through the release of extracellular DNA and associated proteins. Oxidised mitochondrial DNA generated during NETosis has been reported to activate innate immune pathways, including cGAS-STING signalling. In addition, monocytes have increasingly been recognised as important contributors to SLE pathogenesis, and cGAS-STING signalling has emerged as a key pathway capable of inducing type I IFN responses in monocytes. However, whether NET-associated DNA circulating in SLE plasma may directly activate cGAS-STING signalling in human monocytes and contributes to type I IFN responses remains unclear.

**Added value of this study**

Using longitudinal plasma samples from patients with SLE, together with *in vitro*-generated NETs and primary human monocytes, we demonstrate that NET-associated DNA has the capacity to activate the cGAS-STING pathway and induce type I IFN responses. Circulating MPO-DNA levels were associated with plasma IFNβ concentrations, and SLE plasma induced STING and TBK1 phosphorylation as well as IFNβ expression in healthy donor monocytes in a disease activity-dependent manner. *In vitro*-generated NETs recapitulated these responses, inducing cGAS-STING activation and IFNα/IFNβ production, which were reduced by cGAS inhibition. Furthermore, our findings reveal distinct patterns of cytokine induction by intact NETs and DNA-depleted NETs, suggesting that intact NET structures and DNA-digested NET preparations elicit distinct monocytes-derived cytokine responses.

**Implications of all the available evidence**

Together with previous studies demonstrating the inflammatory potential of NET-derived nucleic acids, our findings identify NET-associated DNA in SLE plasma as a potential endogenous trigger of cGAS-STING-mediated IFNβ responses in human monocytes. These results provide new mechanistic insight into how neutrophil-derived extracellular DNA may contribute to sustained type I IFN inflammation in SLE. Furthermore, these findings establish a foundation for understanding how the molecular features and intracellular processing of NET-associated DNA shape innate immune activation, and support exploration of NET-associated DNA sensing through the cGAS-STING pathway in monocytes and its potential relevance to therapeutic targeting in SLE.

## Introduction

The cyclic GMP-AMP synthase (cGAS)-stimulator of interferon genes (STING) signalling pathway is a central component of the innate immune system responsible for sensing cytosolic double-stranded DNA (dsDNA) derived from pathogenic or endogenous sources. Upon dsDNA recognition, cGAS undergoes phase separation to form microcondensates that concentrate cGAS and DNA, thereby facilitating the synthesis of cyclic GMP-AMP (cGAMP),^1^^,^^2^ which activates STING and initiates downstream signalling through TANK-binding kinase 1 (TBK1) and interferon regulatory factor 3 (IRF3).^3^, ^4^, ^5^, ^6^ This cascade culminates in the production of type I interferons (IFNα and IFNβ) and pro-inflammatory cytokines, orchestrating a robust antiviral and antimicrobial response while maintaining immune homoeostasis.^7^^,^^8^ While essential for host defence, dysregulation of cGAS-STING signalling can lead to aberrant immune activation and has been increasingly implicated in autoimmune and inflammatory diseases.^9^

Systemic lupus erythematosus (SLE) exhibits a prominent type I interferon signature driven in part by chronic exposure to self-derived DNA. Sources of self-DNA include defectively cleared apoptotic cells, tissue damage, and retention of neutrophil extracellular traps (NETs). NETs are extracellular DNA-protein structures released by activated neutrophils that contribute to host defence by trapping and neutralising pathogens.^10^, ^11^, ^12^ In SLE, excessive NET formation and impaired clearance increase the availability of extracellular self-DNA, promoting activation of DNA-sensing pathways and perpetuating autoimmune responses.^13^, ^14^, ^15^, ^16^ Persistent stimulation of DNA-sensing pathways, such as cGAS-STING, promotes both innate and adaptive immune responses, contributing to the breakdown of immunological tolerance and disease progression. Whether and how the activation of cGAS-STING contributes to SLE disease progression is not yet fully understood.

Type I IFN production in SLE has been attributed primarily to plasmacytoid dendritic cells (pDCs) responding to immune complexes via Toll-Like Receptors (TLRs).^17^ However, monocytes have been shown to sustain IFNα level in SLE.^18^, ^19^, ^20^

Accumulating evidence from patient studies and experimental models indicates that monocytes play a critical role of SLE pathogenesis^21^^,^^22^ with monocyte lineages are predominantly responsible for STING-mediated pro-inflammatory responses.^19^^,^^23^, ^24^, ^25^ Despite these findings, the endogenous sources of immunostimulatory DNA responsible for activating cGAS-STING signalling in human monocytes remain poorly defined.

Given the abundance of NETs in the plasma of patients with SLE and the ability of monocytes to maintain type I IFN responses and its relation to disease severity, we hypothesised that NET-associated DNA can activate the cGAS-STING pathway in monocytes. We therefore investigated whether NET-associated DNA in SLE plasma induces cGAS-STING pathway activation in monocytes and whether this response is associated with disease activity. Defining this mechanism may provide insight into how NET-derived DNA triggers monocyte-driven type I interferon responses, and how NET-associated DNA, monocyte signalling, and inflammatory responses are related in SLE.

## Methods

### Recruitment of participants

Peripheral monocytes and plasma samples were collected from nine healthy donors through the Mini Donor Service (MDS) at the University Medical Center (UMC) Utrecht, blood samples were collected and stored in sodium citrate tubes. All participants gave written informed consent and the MDS operates in compliance with the ethical guidelines from the World Medical Association's Declaration of Helsinki.

SLE plasma samples were collected from nine patients in remission and nine patients with active SLE as part of the Prediction Of Flares In Lupus with autoantibodiEs and chemokines (PROFILE) study. This longitudinal study tracked patients over a two-year period, with blood samples collected in sodium citrate tubes every three months, yielding a total of nine time points per patient, one patient contributed eight samples as the final time point was unavailable. For patients with active SLE, samples from all-time points were analysed (80 samples); whereas for patients in remission, only the midpoint (12-month) sample was included (nine samples). Individuals diagnosed with antiphospholipid syndrome (APS) were excluded from the selection.

Healthy donors and patients with quiescent SLE were selected based on age and sex to match the active disease cohort. Biological sex and ethnicity were recorded in the electronic case report form (eCRF) and transferred to the study database; the female-only composition reflected the eligible plasma samples available after screening and was not a predefined sex-based inclusion criterion, while ethnicity terminology was updated to contemporary, respectful terminology without reclassification. Additional clinical characteristics of the included participants are provided in Table S1.

### Isolation, culture and stimulation of primary monocytes

Peripheral blood from healthy donors was collected into sodium citrate-coated tubes. Blood was diluted 1:1 with Dulbecco's Phosphate-Buffered Saline (DPBS; Sigma, Cat. No. D8537) and layered carefully over Ficoll–Paque™ PLUS (Cytiva, Cat. No. 10354252) to isolate peripheral blood mononuclear cells (PBMCs) via density gradient centrifugation. Monocytes were subsequently enriched using an autoMACS Separator (Miltenyi Biotec, Cat. No. 130092545) according to the manufacturer's protocol. Negative selection was performed using the Pan Monocyte Isolation Kit (Miltenyi Biotec, Cat. No. 130096537).

Cells were cultured in RPMI 1640 medium (Gibco, Cat. No. 52400025) supplemented with 10% (v/v) fetal bovine serum (FBS; Capricorn Scientific, Lot. No. CP247236), 1% (v/v) penicillin-streptomycin solution (Merck, Cat. No. P0781100ML), and 1% (v/v) L-glutamine (Thermo Fisher Scientific, Cat. No. 25030024). Cultures were maintained at 37°C in a humidified 5% CO_2_ atmosphere.

Monocytes were cultured for 24 h in culture medium containing tenfold-diluted patient plasma (1:10 dilution).^26^ Monocytes were stimulated with NETs at a DNA concentration of 200 ng/mL. Separately, DNase-digested NETs (deNETs) were added in equivalent volumes, yielding a DNA concentration of approximately 72.59 ng/mL. For blockade of type I interferons signalling, monocytes were treated with 10 μg/mL anifrolumab (Saphnelo, AstraZeneca).^27^, ^28^, ^29^, ^30^ For inhibition of cGAS activity, monocytes were pre-treated with 10 μM cGAS-IN-1 (MedChemExpress, Cat. No. HY-149526_5MG).^31^

### Preparation of NETs and deNETs

Neutrophils were isolated from EDTA-anticoagulated whole blood by Ficoll–Paque™ PLUS density gradient centrifugation, followed by erythrocytes depletion using hypotonic lysis. The isolated neutrophils were resuspended in phenol red-free DMEM/F-12 medium (Gibco, Cat. No. 11039021) and distributed into tissue culture flasks (Greiner Bio-One, Cat. No. 658175) for 30 min at 37°C to allow adherence.

Platelets from the same donors were activated with lipopolysaccharide (LPS) from *Escherichia coli* serotype O111:B4 (Merck, Cat. No. LPS25) at 1 × 10^5^ EU/mL for 1 h at 37°C and subsequently overlaid onto the neutrophil monolayer for 60 min to induce NET formation. After co-culture, supernatants were removed, and adherent neutrophils were washed twice with PBS.^32^

Chromatin was solubilised using micrococcal nuclease (Thermo Fisher Scientific, Cat. No. 88216) at 5 U/mL. Supernatants were cleared by two sequential centrifugation steps to remove remaining cellular material and insoluble fragments, then collected for later analyses. NETs were prepared independently from twelve healthy donors and pooled for DNA quantification and as source for DNase-digested NETs (deNETs).

For deNETs preparation, NET suspensions were treated with DNase I (STEMCELL Technologies, Cat. No. 07900; 1 mg/mL, ≥2000 U/mL) at 40 U/mL for 30 min at 37°C. DNase activity was terminated by adding EDTA to a final concentration of 5 mM, followed by gentle mixing. Undigested NETs and medium were adjusted to the same EDTA concentration as deNETs before monocyte stimulation.^15^

### Quantification of dsDNA in plasma, NETs and deNETs

NET-associated extracellular DNA was stained with SYTOX™ Orange (Invitrogen, Cat. No. 10338062) at a final concentration of 0.2 μM, followed by 20 min incubation at 4°C in the dark. Fluorescence was measured using a CLARIOstar microplate reader (BMG Labtech) with excitation/emission wavelengths of 530 ± 15 nm and 645 ± 20 nm, respectively. DNA concentrations were interpolated from a standard curve generated using serial dilutions of purified human genomic dsDNA (Promega, Cat. No. G3041). NET preparations contained 843.24 ng/mL DNA, whereas deNETs contained 305.86 ng/mL.

### Quantification of plasma MPO-DNA complexes (NET-associated DNA)

96-well MaxiSorp plates (Fisher Scientific, Cat. No. 439454) were coated with 100 μL of 5 μg/mL anti-myeloperoxidase (MPO) antibody (Bio-Rad, Cat. No. 0400-0002) in PBS and incubated at 4°C overnight. Plates were washed three times with PBS containing 0.05% Tween-20 and subsequently blocked with the same buffer supplemented with 1% bovine serum albumin (BSA) for 1 h at room temperature. After washing, 20 μL of undiluted plasma was added to each well together with 80 μL of anti-DNA-peroxidase (POD) conjugate (Cell Death Detection ELISAPLUS, Sigma–Aldrich, Cat. No. 11774425001) prepared as a 1:20 dilution in incubation buffer. Plates were incubated for 2 h at room temperature with continuous shaking at 300 rpm. Following incubation, unbound conjugate was removed by washing, and wells were then incubated with 100 μL of ABTS substrate solution. Signal was monitored kinetically at 405 nm at 1-min intervals for 30 min using a SpectraMax M3 microplate reader (Molecular Devices LLC, California, USA).

### Measurement of plasma citrullinated histone H3 (citH3)

Plasma citrullinated histone H3 (citH3) levels were quantified using the Citrullinated Histone H3 (Clone 11D3) ELISA Kit (Cayman Chemical, Cat. No. 501620) according to the manufacturer's instructions. Plasma samples were diluted 2.5-fold in assay buffer and added in to the pre-coated 96-well ELISA plate. After incubation for 2 h at room temperature on an orbital shaker, wells were washed five times with wash buffer and incubated with anti-histone H3 horseradish peroxidase (HRP)-conjugated antibody for 1 h at room temperature. Following an additional washing step, 100 μL of TMB substrate solution was added to each well, and plates were incubated for 30 min at room temperature in the dark. The reaction was terminated by adding 100 μL of HRP stop solution, and absorbance was measured at 450 nm and 570 nm using CLARIOstar microplate reader (BMG Labtech).

### Multiplex immunoassay

Plasma samples and supernatants from monocytes stimulated for 48 h were analysed for cytokine profiles using the LEGENDplex™ Human Inflammation Panel 1 (BioLegend, Cat. No. 740808) and the LEGENDplex™ Human Anti-Virus Response Panel 1 (BioLegend, Cat. No. 741269) according to the manufacturer's instructions. Samples were incubated with fluorescent beads coated with cytokine-specific capture antibodies, followed by incubation with biotinylated detection antibodies and streptavidin-PE. Data were acquired by flow cytometry, and cytokine concentrations were calculated using standard curves provided with each kit.

### Flow cytometry

Cells (2 × 10^5^ per sample) were washed once with PBS and stained with eFluor 780 viability dye (1:1000; eBioscience, Cat. No. 65086518) for 15 min at 4°C in the dark. Cells were subsequently fixed and permeabilized using a 1:3 mixture of Fixation/Permeabilization Concentrate (eBioscience, Cat. No. 00512343) and Fixation/Permeabilization Diluent (eBioscience, Cat. No. 00522356) for 30 min at 4°C in the dark. Following permeabilization, cells were washed with 1 × Permeabilization Buffer (eBioscience, Cat. No. 00833356, diluted 1:10 in deionised water), and stained with antibody mixtures prepared in 1 × Permeabilization Buffer for 30 min at 4°C in the dark. Cells were washed twice with 1 × Permeabilization Buffer. Finally, cells were resuspended in FACS buffer (PBS + 1% BSA + 0.1% sodium azide).

Flow cytometric acquisition was performed using BD LSRFortessa™ Cell Analyser (BD Biosciences, Cat. No. 647794), data were collected and analysed using BD FACSDiva software.

Research Resource Identifiers (RRIDs) for the antibodies used in this study are provided in Table S3. Their validation for use in flow cytometry was checked using the RRID Portal and/or manufacturer product pages.

### Ethics

The study was approved by the Medical Research Ethics Committee NedMec, Utrecht, the Netherlands (protocol number NL75276.041.21). All participants provided written informed consent. The study was conducted in accordance with the Declaration of Helsinki (2024).

### Statistics

Data analysis and visualisation were performed using Microsoft Excel (version 2302), the Qognit BioLegend online platform, GraphPad Prism (version 10.1.2), FlowJo (version 10.8.1), Cytoscape 3.10.4, the web-based Morpheus platform (Broad Institute; RRID:SCR_017386) and Adobe Illustrator (2022).

Due to the relatively small sample sizes of individual groups and biological variability inherent to the datasets, non-parametric statistical tests were used throughout the study. Two-group comparisons were performed using the Wilcoxon matched-pairs signed rank test (paired) or the Mann–Whitney U test (unpaired). For multiple comparisons, the Friedman test was applied to paired datasets, and the Kruskal–Wallis test to unpaired datasets. Correlations between variables were assessed using Spearman's rank correlation analysis. Spearman correlation coefficients (ρ) and P-values were reported, with 95% confidence intervals displayed where indicated.

Sample sizes were determined by the availability of eligible clinical samples and healthy donors; no formal a priori sample size calculation was performed. Randomisation was not applicable, as clinical samples were analysed according to predefined disease activity groups and experimental conditions. No formal blinding was implemented; however, experimental measurements and statistical analyses were performed independently of access to individual patient clinical records. Inclusion and exclusion criteria for the clinical cohort are described in the Recruitment of Participants section, and samples were included in each analysis according to their availability at the time of the respective experiment.

### Role of funders

No funders had any role in the study design, data collection, data analysis, interpretation of the results, writing or revision of the article.

## Results

### Type I interferon responses are associated with SLE disease activity and circulating NET-associated markers

To determine whether circulating cell-free DNA (cfDNA), neutrophil extracellular trap (NET)-associated markers (MPO-DNA complex and citrullinated histone H3 (CitH3)), and type I, II, III interferons (IFNs) are altered in systemic lupus erythematosus (SLE), we examined plasma samples from nine patients with active disease followed longitudinally at three-month intervals over two years (80 samples); together with nine patients with quiescent SLE and nine healthy donors (Table S1). Samples from patients with active SLE were stratified according to the Systemic Lupus Erythematosus Disease Activity Index (SLEDAI) into low (0–4), moderate, (5–11) and high (≥12) disease activity groups.

Quantitative analysis revealed that circulating DNA concentrations were markedly increased in patients with active SLE compared to both healthy controls and patients in remission (P < 0.05, Fig. 1A). In addition, plasma collected from patients with high disease activity (SLEDAI ≥12) contained higher levels of MPO-DNA complexes than samples from patients with low disease activity (SLEDAI ≤4) (P < 0.05, Fig. 1B). Plasma CitH3 levels showed a similar trend, although this did not reach statistical significance (P = 0.059, Fig. 1C).

**Fig. 1:**
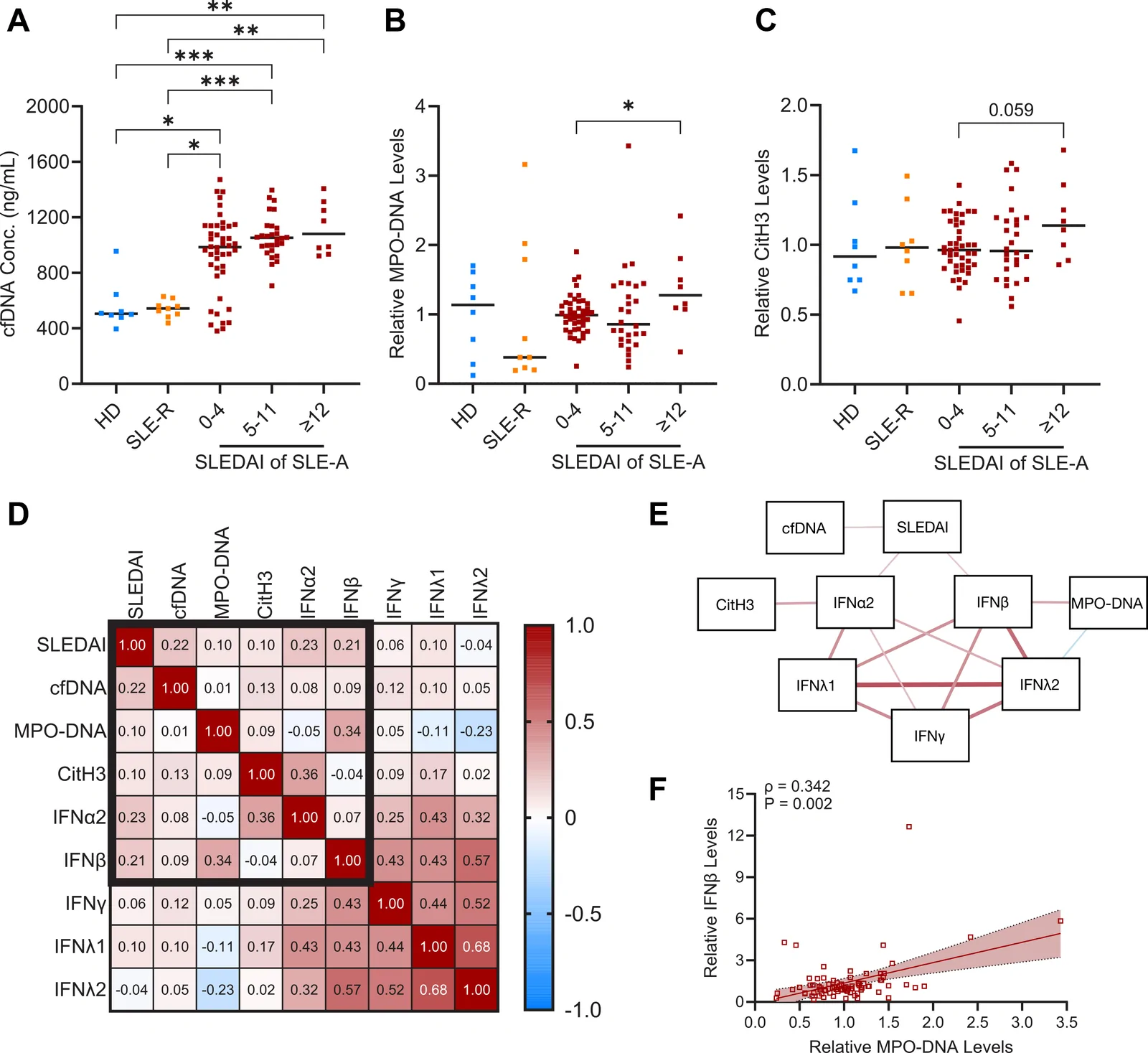
**Circulating NET-associated markers and type I interferons are elevated during periods with high SLE disease activity, with MPO-DNA associated with IFNβ. Longitudinal analysis of plasma DNA, circulating NETs, disease activity, and interferon levels in patients with SLE.** Eight healthy donors (HD), nine patients in remission (SLE-R), and nine patients with active disease (SLE-A) were included, with groups matched for age and sex. Patients with SLE were followed for up to 2 years; for remission case, sample at the median 12-month time point was analysed, while all available time points for active patients were included, yielding a total of 80 plasma samples. A. The concentration of cell-free DNA (cfDNA) in plasma was quantified using Sytox Orange fluorescence, and values presented as medians. Plasma MPO-DNA (B) and CitH3 (C) levels were measured by ELISA and are presented as relative levels. For both assays, HD and SLE-R samples were normalised to the mean ELISA signal of their respective groups, whereas SLE-A samples were normalised to each patient's individual baseline, defined as the average of all samples collected during periods of low disease activity (SLEDAI ≤4). A–C: Bars represent median values. HD, n = 8; SLE-R, n = 8 or 9; SLEDAI 0–4, n = 44; SLEDAI 5–11, n = 28; SLEDAI ≥12, n = 8. A: the Kruskal–Wallis test was used for multiple comparisons. B–C: the Mann–Whitney test was used for unpaired comparisons. ∗p < 0.05; ∗∗p < 0.01; ∗∗∗p < 0.001. D. Spearman correlation heatmap showing pairwise correlations among SLEDAI scores, plasma DNA concentrations, MPO-DNA levels, CitH3 levels, and type I, Ⅱ, Ⅲ interferon levels across longitudinal plasma samples (n = 80) from patients with active SLE (n = 9). Longitudinal measurements of NET-associated markers and interferons were normalised within each individual by dividing each value by the baseline measurement obtained when SLEDAI ≤4 to correct for inter-patient variability. E. Correlation network based on Spearman correlation analysis in D. Edges represent correlations with P < 0.1; no cutoff was applied to the correlation coefficient (ρ). Edge intensity is proportional to the absolute Spearman correlation coefficient (|ρ|), and edge colour indicates the direction of correlation as shown in Figure D legend. F. Spearman correlation analysis between plasma MPO-DNA levels and plasma IFNβ concentrations (n = 80).

To assess the association between circulating cfDNA, MPO-DNA, CitH3, IFNs and disease activity, plasma levels of these parameters were integrated with contemporaneous SLEDAI scores (Fig. 1D). This analysis demonstrated that extracellular DNA and type I IFNs (IFNα2 and IFNβ) were associated with SLE disease activity, whereas such associations were not observed for type Ⅱ IFN (IFNγ) or type Ш IFNs (IFNλ1 and IFNλ2). Furthermore, plasma IFNα levels were significantly elevated in patients with severe disease activity (SLEDAI ≥12) compared with healthy donors, patients in remission, and active patients with lower disease activity scores (P < 0.05, Figure S1A). Plasma IFNβ levels were also increased in patients with moderate-to-severe disease activity (SLEDAI 5–11) compared with those with low disease activity (SLEDAI 0–4) (P < 0.05, Figure S1B). Correlation analyses further exhibited positive relationships between SLEDAI scores and IFNα2 (ρ = 0.227, P = 0.043, Figure S1I), while IFNβ levels showed a similar but non-significant trend (ρ = 0.191, P = 0.094, Figure S1J).

Notably, IFNα2 levels did not correlate with MPO-DNA levels but showed a positive correlation with CitH3 levels (Fig. 1D–E; ρ = 0.358, P = 0.001, Figure S1K), whereas IFNβ levels were positively correlated with MPO-DNA levels (ρ = 0.342, P = 0.002, Fig. 1D–F). Collectively, these findings suggest that type I IFN responses are associated with SLE disease activity, and that circulating NET-associated markers are differentially associated with type I IFN responses, with MPO-DNA showing an association with IFNβ and CitH3 showing an association with IFNα2. These findings support further investigation of the capacity of NET-associated DNA to activate innate DNA-sensing pathways.

### Plasma from patients with SLE during periods of increased disease activity is associated with increased IFNβ production by monocytes *ex vivo* and cGAS-STING pathway activation

We next investigated whether plasma containing elevated NET-associated components from patients with active SLE, particularly samples corresponding to higher SLEDAI scores, enhance cGAS-STING pathway activation in healthy monocytes. Peripheral monocytes were isolated from healthy donors and cultured for 24 h in medium containing tenfold-diluted patient plasma.^26^ Following incubation, cells were analysed by flow cytometry (Fig. 2A).

**Fig. 2:**
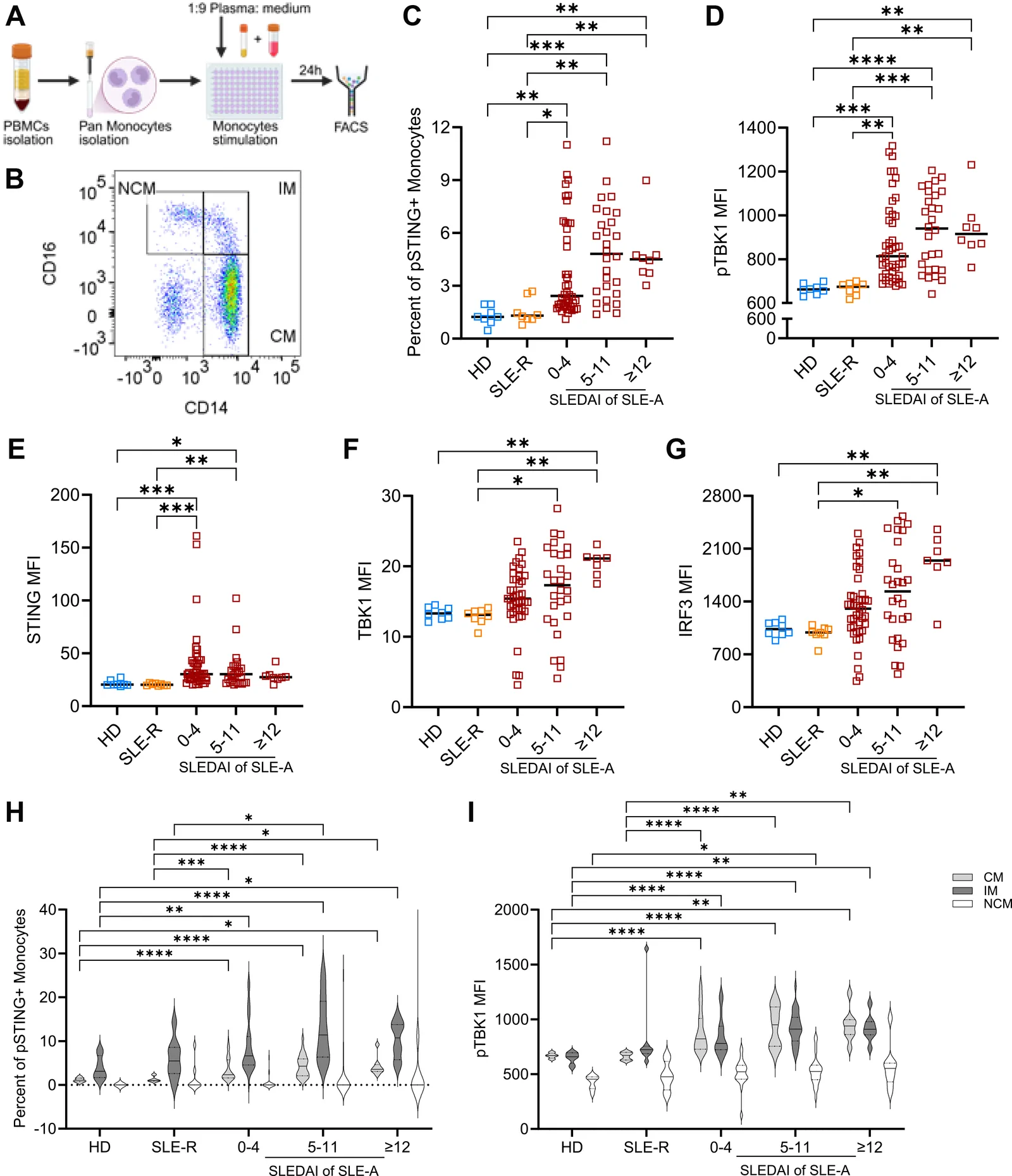
**Plasma from patients with active SLE activates the cGAS-STING pathway in healthy monocytes. Activation of the cGAS-STING pathway in monocytes following stimulation with plasma from patients with SLE and healthy donors.** A. Schematic of the experimental workflow. Plasma samples from healthy donors and patients with SLE were diluted 1:10 and incubated with freshly isolated peripheral blood monocytes for 24 h, followed by flow cytometric analysis of cGAS-STING pathway activation. B. Gating strategy for monocyte subsets, including classical (CM), intermediate (IM), and non-classical (NCM) monocytes, defined by CD14 and CD16 expression. C. Percentage of pSTING-positive monocytes activated by plasma from healthy donors, remission and patients with active SLE, samples from active patients grouped by SLEDAI scores (0–4, 5–11, ≥12). D. MFI of pTBK1 in monocytes activated by plasma. E. MFI of STING in monocytes after plasma stimulation. F. MFI of TBK1 in monocytes after plasma stimulation. G. MFI of IRF3 in monocytes after plasma stimulation. C–G: Bars represent median values. H. Percentage of pSTING-positive monocytes within each subset after stimulation with plasma from five experimental groups. Median (solid line) and quartiles (dotted lines) are indicated. I. MFI of pTBK1 in monocyte subsets following stimulation with plasma from the five groups. Median (solid line) and quartiles (dotted lines) are indicated. MFI represents mean fluorescence intensity. HD, n = 8; SLE-R, n = 8; SLEDAI 0–4, n = 44; SLEDAI 5–11, n = 28; SLEDAI ≥12, n = 8. The Kruskal–Wallis test was used for multiple comparisons. ∗p < 0.05; ∗∗p < 0.01; ∗∗∗p < 0.001; ∗∗∗∗P < 0.0001.

Plasma from patients with active SLE induced significantly higher levels of phosphorylated STING (pSTING) and phosphorylated TBK1 (pTBK1) in healthy primary monocytes compared with plasma from healthy donors or from patients with SLE in remission (P < 0.05, Fig. 2C–D), while cell size and CD14 expression remained unchanged (Figures S2A–B). Furthermore, pSTING (ρ = 0.430, P < 0.0001, Figure S3A) and pTBK1 (ρ = 0.384, P < 0.0004, Figure S3B) expression in monocytes correlated positively with SLEDAI scores of plasma samples from patients with active SLE. Particularly, pTBK1 expression in monocytes is correlated with plasma NET-associated DNA levels (ρ = 0.245, P < 0.029, Fig. 3D).

**Fig. 3:**
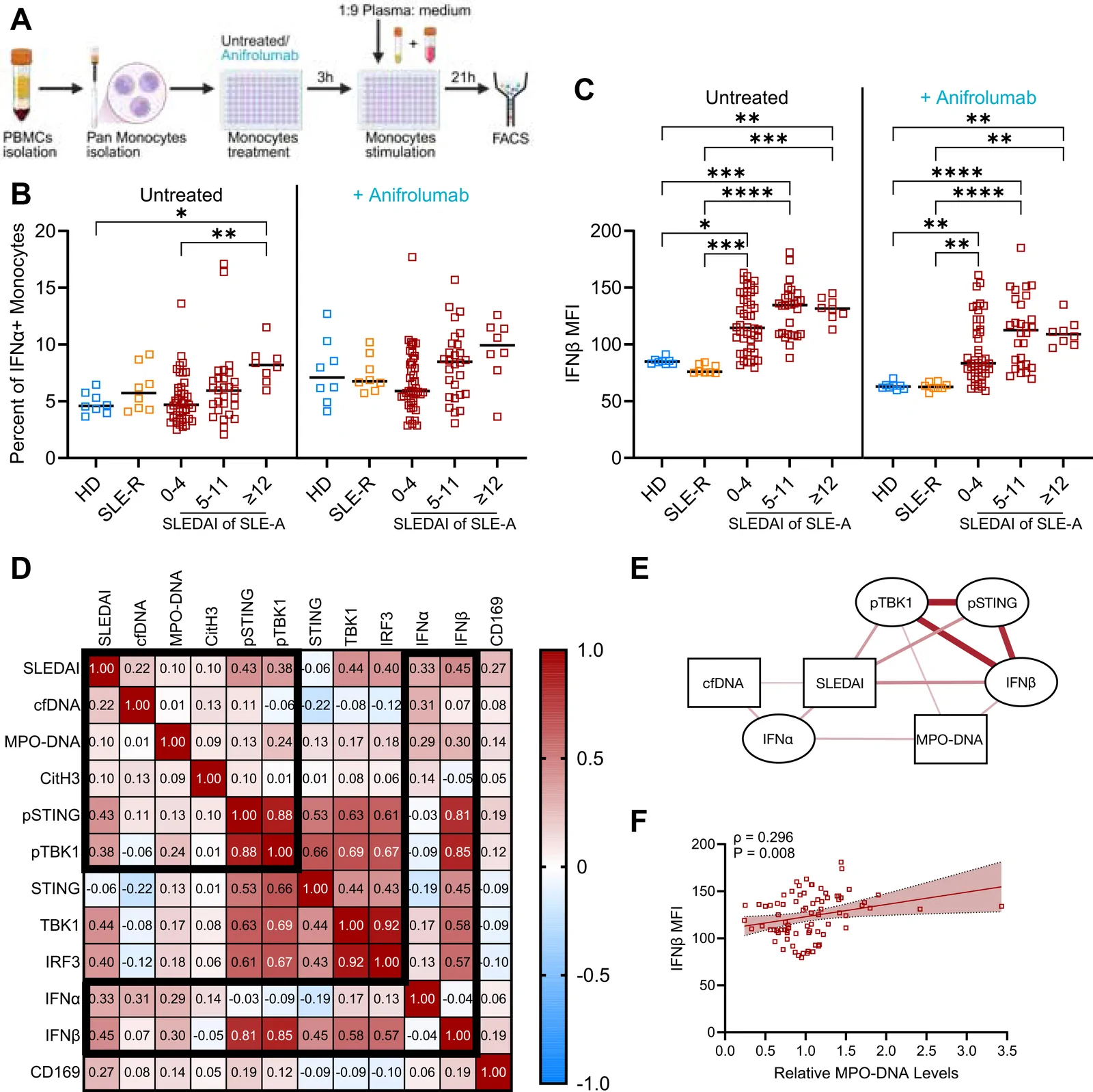
**IFNβ responses induced by SLE plasma are associated with disease activity, persist despite anifrolumab treatment, and correlate with circulating MPO-DNA levels. Effect of circulating NET-associated material on type I interferon induction in monocytes stimulated with SLE plasma.** A. Schematic of the experimental workflow. Primary monocytes isolated from healthy donors were pretreated with anifrolumab for 3 h, followed by stimulation with 1:10 diluted plasma in medium containing the same concentration of anifrolumab for 24 h. Monocytes were subsequently analysed by flow cytometry to assess intracellular IFNα and IFNβ expression. B. Percentage of IFNα-positive monocytes after stimulation with plasma in the absence or presence of anifrolumab. C. MFI of IFNβ in monocytes following plasma stimulation without or with anifrolumab. B–C: Bars represent median values. HD, n = 8; SLE-R, n = 8; SLEDAI 0–4, n = 44; SLEDAI 5–11, n = 28; SLEDAI ≥12, n = 8. The Kruskal–Wallis test was used for multiple comparisons. ∗p < 0.05; ∗∗p < 0.01; ∗∗∗p < 0.001; ∗∗∗∗P < 0.0001. D. Spearman correlation heatmap showing relationships among SLEDAI scores, plasma cfDNA concentrations, MPO-DNA levels, CitH3 levels in SLE samples, and monocyte responses, including pSTING, pTBK1, STING, TBK1, IRF3, IFNα (with anifrolumab treatment), IFNβ, and CD169 expression (n = 80). E. Correlation network based on Spearman correlation analysis in D. Only significant correlations (P < 0.05) with an absolute Spearman correlation coefficient (|ρ| > 0.1) are displayed. Edge intensity represents the absolute correlation coefficient (|ρ|), and edge colour indicates the direction of correlation as shown in Figure D legend. F. Spearman correlation between intracellular IFNβ MFI in monocytes and plasma MPO-DNA levels in active SLE samples (n = 80). MFI, mean fluorescence intensity.

Total expression of STING, TBK1, and IRF3 was increased in monocytes stimulated with plasma samples from patients with higher SLEDAI scores (P < 0.05, Fig. 2E–G; Fig. 3D). While these pathway components (ρ = 0.450, P < 0.0001, Figure S3E; ρ = 0.579, P < 0.0001, Figure S3G; ρ = 0.573, P < 0.0001, Figure S3D) showed associations with IFNβ production, pSTING (ρ = 0.813, P < 0.0001, Figure S3D) and pTBK1 (ρ = 0.853, P < 0.0001, Figure S3F) displayed stronger correlations, suggesting that cGAS-STING signalling activity contributes to IFNβ induction. To further define the cellular context of cGAS-STING activation, we analysed pathway activation across monocyte subsets. We found that pSTING (P < 0.05, Fig. 2H) and pTBK1 (P < 0.05, Fig. 2I) expression was significantly increased in classical and intermediate monocytes from patients with higher disease activity, suggesting that inflammatory monocyte subsets may contribute to cGAS-STING-mediated immune activation in response to circulating factors, including NET-associated DNA, in active SLE.

CD169 (SIGLEC1) expression on monocytes enhanced following stimulation with plasma from patients with higher disease activity (P < 0.05, Figure S2C; Fig. 3D), supporting the downstream type I IFN responses. To assess *de novo* type I IFN production by monocytes, we stimulated healthy primary monocytes with plasma from patients with SLE in the presence of anifrolumab to block IFNAR1-mediated signalling (Fig. 3A). We found that the increased IFNα signal in monocytes stimulated with plasma samples from patients with higher SLEDAI scores was diminished following culture in the presence of anifrolumab (P < 0.05, Fig. 3B), suggesting that a substantial proportion of the elevated intracellular IFNα signal observed after SLE plasma stimulation is influenced by IFNAR1-mediated type I IFN signalling rather than representing a fully IFNAR1-independent response.

In contrast, the increase in IFNβ expression under the same conditions remained largely unchanged (P < 0.05, Fig. 3C), and IFNβ expression presented strong correlation with pSTING and pTBK1 activation (Fig. 3D–E; Figure S3D–H). In addition, NET-derived DNA levels of patients’ plasma showed a positive correlation with IFNβ expression in monocytes (ρ = 0.296, P = 0.008, Fig. 3F). Together, these findings demonstrate that IFNβ production by monocytes upon plasma stimulation supports the contribution of the cGAS-STING pathway.

### *In vitro* generated NETs activate the cGAS-STING signalling pathway and promote type I IFN production by monocytes

Building on our plasma stimulation experiments showing that plasma from patients with higher disease activity induces cGAS-STING activation and IFNβ production in monocytes, and that plasma MPO-DNA levels are associated with both circulating IFNβ levels and IFNβ expression in monocytes following plasma stimulation, we next sought to directly assess whether *in vitro* generated NETs drive cGAS-STING activation and type I IFN production through intracellular pathways in human monocytes. To this end, monocytes were stimulated with *in vitro* produced NETs or DNase-digested NETs (DeNETs), which contain substantially reduced DNA compared with intact NETs. The concentration of NET-derived DNA used for stimulation was selected based on the range of MPO-DNA levels detected in diluted SLE plasma samples. Given recent evidence identifying monocytes as a major source of STING-mediated IFNα production, we assessed cGAS-STING pathway in the presence or absence of cGAS inhibitor cGAS-IN-1 by measuring intracellular pSTING, pTBK1, type I IFNs at 24 h post stimulation using flow cytometry, and evaluated IFNα and IFNβ secretion at 48 h using multiplex immunoassays (Fig. 4A).

**Fig. 4:**
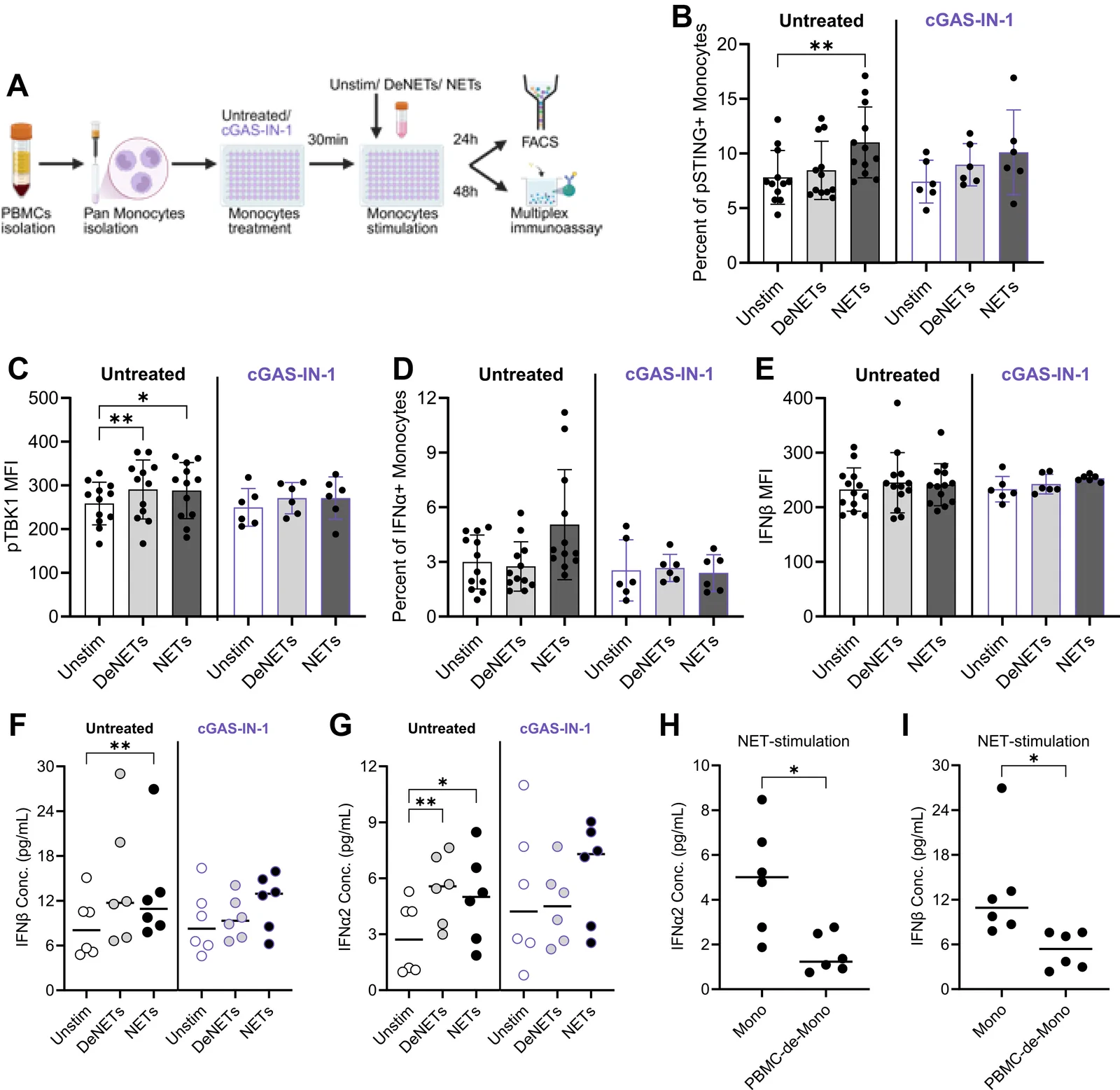
***In vitro*-generated NETs activate cGAS-STING signalling and induce type I interferon production in monocytes. Exposure to NETs triggers activation of the cGAS-STING pathway and type I IFN production amongst monocytes in PBMC, as induced by depletion experiments using DNase-treated NETs, cGAS signalling inhibitor and depletion of monocytes from PBMC.** A. Experimental workflow. Primary monocytes isolated from healthy donors were pretreated with medium or cGAS-IN-1 for 30 min and subsequently stimulated with medium (unstimulated control), DNase-digested NETs (DeNETs), or intact NETs at equivalent volumes. After 24 h, half of the cells were collected for flow cytometric analysis, while the remaining cells were cultured for an additional 24 h before supernatants were collected for multiplex immunoassay analysis. B. Percentage of pSTING-positive monocytes following treatment and stimulation for 24 h (Untreated, n = 12; cGAS-IN-1 pre-treated, n = 6). C. MFI of pTBK1 in monocytes following treatment and stimulation for 24 h (Untreated, n = 12; cGAS-IN-1 pre-treated, n = 6). D. Percentage of IFNα-positive monocytes following treatment and stimulation for 24 h. E. MFI of intracellular IFNβ in monocytes following treatment and stimulation for 24 h (Untreated, n = 12; cGAS-IN-1 pre-treated, n = 6). B–E: Bars represent mean values, with error bars indicating standard deviation. F. IFNα2 concentration in monocyte supernatants following treatment and stimulation for 48 h (n = 6). G. IFNβ concentration in monocyte supernatants following treatment and stimulation for 48 h (n = 6). H. IFNα2 secretion from monocytes compared with an equal number of monocyte-depleted PBMCs from the same donors following NET stimulation for 48 h (n = 6). I. IFNβ secretion from monocytes compared with an equal number of monocyte-depleted PBMCs from the same donors following NET stimulation for 48 h (n = 6). F–I: Bars represent median values. MFI, mean fluorescence intensity. Within each treated and stimulated condition, the Friedman test with Dunn's multiple comparisons test was used for multiple comparisons. ∗p < 0.05; ∗∗p < 0.01.

Stimulation with intact NETs, but not DeNETs, induced phosphorylation of STING and this response was inhibited by cGAS-IN-1 (P < 0.01, Fig. 4B), whereas phosphorylation of TBK1 increased under both NETs and DeNETs stimulation (P < 0.05, Fig. 4C). The ability of DeNETs to elevate pTBK1 in monocytes suggests that NET immunostimulatory activity may not depend solely on large DNA structures. Residual DNA fragments and/or NET-associated proteins may contribute by facilitating nucleic acid uptake, intracellular trafficking, or activation of innate immune pathways.

Total STING expression remained unchanged in monocytes following NET stimulation compared with unstimulated controls (Figure S4C). Intracellular levels of IFNα and IFNβ measured by flow cytometry were not affected by either NET or DeNET stimulation (Fig. 4D–E). At the level of secretion, both NETs and DeNETs induced secretion of IFNα and inhibited by cGAS-IN-1 (P < 0.05, Fig. 4F), whereas IFNβ secretion was significantly increased only in response to intact NETs when cGAS-IN-1 was absent (P < 0.01, Fig. 4G), consistent with the preferential IFNβ response and its association with cGAS-STING activation observed following stimulation with plasma from patients with higher SLEDAI scores (Fig. 3D–F).

To further define the cellular source of type I interferons, we compared IFNα and IFNβ secretion between equal numbers of purified monocytes and monocyte-depleted peripheral blood mononuclear cells from the same donors. Both IFNα and IFNβ levels were markedly higher in supernatants from purified monocytes than from monocyte depleted PBMCs (P < 0.05, Fig. 4H–I), indicating that monocytes were the predominant contributors to NET induced type I IFN production under the experimental condition.

### NETs and DeNETs induce distinct inflammatory cytokine signatures and differential type I interferon associations in SLE

Besides type I interferons, monocytes may produce additional pro-inflammatory cytokines in response to stimulation with NETs and DeNETs, potentially contributing to the inflammatory environment observed in SLE. To investigate whether NETs and DeNETs modulate the secretion of cytokines reported to be elevated or dysregulated in SLE, we measured a panel of immunomodulatory cytokines in monocyte culture supernatants at 48 h. We further examined their association with IFNβ secretion to determine whether these cytokine responses showed a pattern consistent with cGAS-STING pathway activation.

We found that in addition to secreting IFNβ, NET stimulation induced markedly higher secretion of GM-CSF, TNFα, CCL2, IL1β, IL6, IL8 and IL10 compared with DeNETs (Fig. 5A–B), indicating that intact NET-associated structures elicited a broader inflammatory cytokine response than fragmented NET material. Previous studies have shown that cGAS-STING activation can induce inflammatory mediators, including IL-6,^33^, ^34^, ^35^ IL-8^24^ and IL-10^23^^,^^36^ in monocytes, which are also among the cytokines preferentially induced by intact NETs compared with DeNETs in our study.

**Fig. 5:**
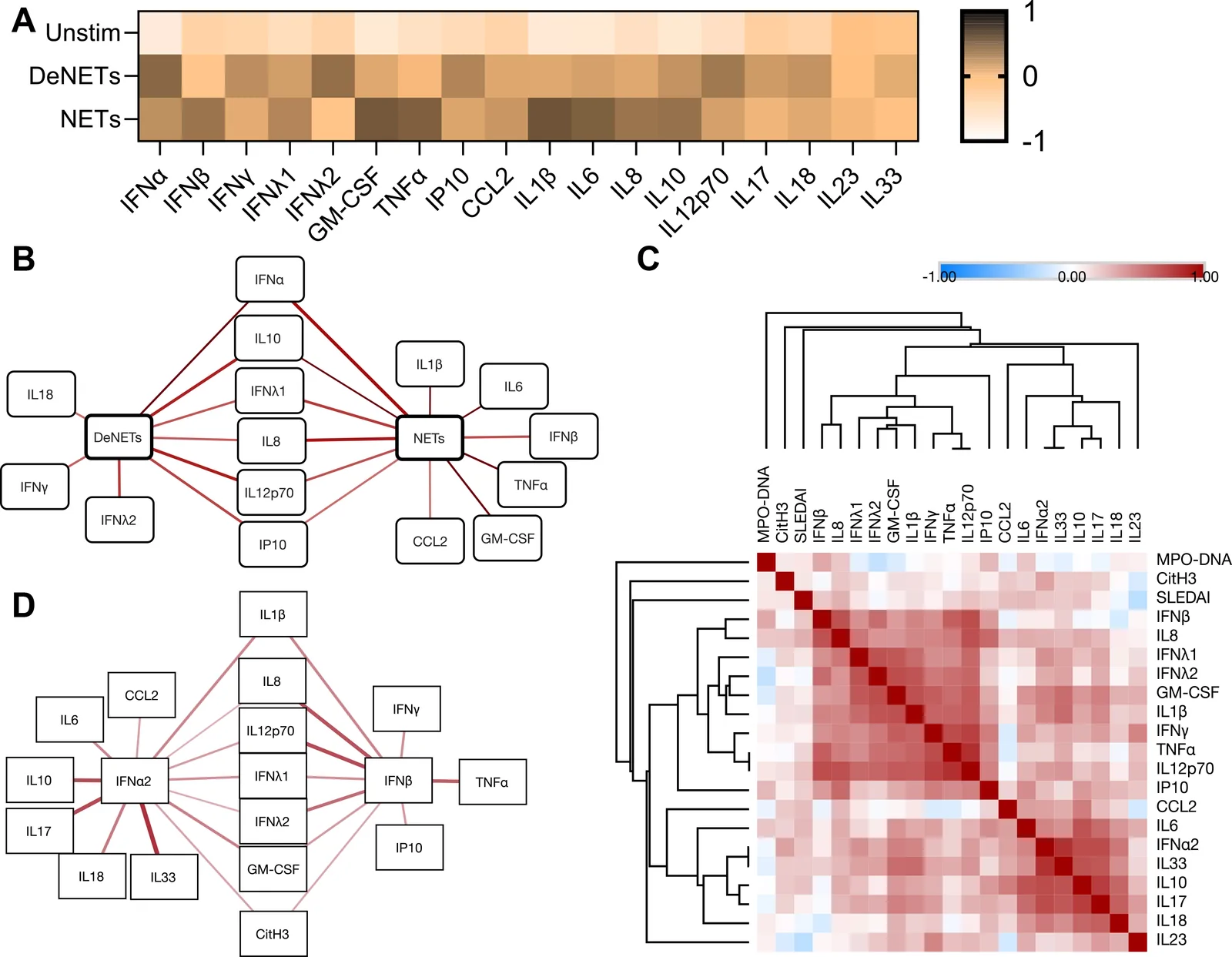
**NET-associated components differentially regulate type I interferon and inflammatory cytokine responses. Distinct cytokine responses induced by NETs and DNase-digested NETs and their association with inflammatory networks in SLE.** A. Heatmap showing relative cytokine responses in monocytes across three stimulation conditions (unstimulated, DeNETs, intact NETs; n = 6). Z-scores were calculated across the three stimulation conditions for each donor and summarised across donors using the median. Positive values indicate cytokine levels above the median, whereas negative values indicate levels below the median, highlighting relative changes between stimulation conditions. B. Network representation of cytokines preferentially induced by DeNETs or NETs (n = 6). Cytokine responses are shown as changes in normalised Z-scores relative to unstimulated controls. For each cytokine, ΔZ was calculated as Z(stimulated)-Z(unstimulated). Cytokines showing sufficient induction (ΔZ > 0.5) following either DeNETs or NETs stimulation were included in the corresponding cluster. Cytokines with similar induction between the two stimuli (|ΔΔZ| ≤ 0.3, where ΔΔZ = ΔZ(DeNETs)-ΔZ(NETs)) were considered shared responses and connected to both clusters. Cytokines with |ΔΔZ| > 0.3 were assigned to the stimulus producing the stronger response. Edge thickness represents the magnitude of cytokine induction (ΔZ) relative to unstimulated controls. C. Hierarchical clustering of the Spearman correlation matrix to show a cytokine network characterised by strong associations among type I interferon-related mediators and inflammatory cytokines measured in active SLE plasma (n = 80). D. Correlation network showing relationships among cytokines measured in active SLE plasma (n = 80). Only significant correlations (P ≤ 0.05) with an absolute Spearman correlation coefficient (|ρ| ≥ 0.30) are displayed. Edge thickness reflects the strength of correlation based on |ρ|, and edge colour indicates the direction of correlation as shown in the legend of C.

Given our observation that IFNα and IFNβ responses were differentially regulated following NET stimulation, we next investigated whether these distinct cytokine patterns were reflected in SLE plasma. We performed a hierarchical clustered heatmap based on Spearman correlation coefficients (ρ) among circulating cytokines, NET-associated markers, and SLEDAI record in active SLE plasma (Fig. 5C). This analysis revealed distinct correlation patterns, with IFNα2 and IFNβ segregating into different correlation clusters with other inflammatory mediators. To further visualise these relationships, we generated a correlation network highlighting cytokines associated with IFNα- and IFNβ-related inflammatory signatures (Fig. 5D). Plasma CCL2, IL6, IL10, IL17, IL18 and IL33 clustered with IFNα2; whereas IL-8, IL12p70, IP10, TNFα and IFNγ clustered with IFNβ (Fig. 5C–D).^23^^,^^36^ The individual correlations between IFNβ and the associated inflammatory markers are shown in Figure S5.

Take together, these results support an association between NET-associated DNA, cGAS-STING activation and IFNβ production by monocytes in SLE, with these responses being more pronounced at higher disease activity, and provide experimental evidence that NET-associated DNA can promote monocyte-mediated inflammatory responses through cGAS-STING activation.

## Discussion

Systemic lupus erythematosus (SLE) is featured with dysregulated nucleic acid sensing and persistent interferon signalling. Here, we demonstrate that circulating DNA, NET-associated markers, and type I interferons correlate with disease activity, with IFNβ displaying a stronger association with NET-associated DNA than IFNα. We further identified that SLE plasma activates the cGAS-STING pathway and induce IFNβ production in human monocytes *ex vivo*, with pSTING, pTBK1, and IFNβ expression in monocytes associated with the SLEDAI of the plasma donors (Figure S3A–C); while *in-vitro*-generated NETs can also activate this pathway and promote IFNβ secretion, providing experimental evidence for NET-associated DNA sensing through cGAS-STING in monocytes.

Although NETs have been implicated in SLE pathogenesis, circulating MPO-DNA and CitH3 represent indirect markers of NET biology and are influenced by NET formation, degradation, clearance, and tissue redistribution. Consequently, circulating NET-associated markers may not accurately reflect the immunostimulatory potential of NETs. Our functional experiments nevertheless demonstrate that NET-derived DNA can activate the cGAS-STING signalling and induce IFNβ production in monocytes, raising the possibility that the biological activity of NET-derived nucleic acids may contribute to type I interferon responses in SLE independently of their absolute circulating abundance, a hypothesis that warrants further investigation.

Our *in vitro* experiments further suggest that intact NET structure preferentially promote IFNβ production, whereas both NETs and DeNETs stimulate IFNα secretion, consistent with a greater dependence of IFNβ responses on intact NET-associated DNA and cGAS-STING signalling.^3^ These findings are consistent with the concept that efficient cGAS-STING activation depends on the structural integrity and intracellular delivery of NET-associated DNA, while IFNα may involve additional endosomal DNA-sensing pathways. Uptake of intact NET structures or NET fragments by monocytes may facilitate intracellular delivery of NET-associated DNA through phagocytic internalisation and potential escape from endosomal/phagosomal compartments to access cytosolic cGAS, whereas fragmented DNA generated following DNase digestion may preferentially engage endosomal sensors. Consistent with this model, plasma IFNβ correlated with NET-associated DNA but not total circulating DNA, supporting a potential link between intact NET-derived DNA and cGAS-STING-dependent IFNβ production.

Beyond interferon induction, NET stimulation elicited a broader spectrum of pro-inflammatory and immunomodulatory cytokines, consistent with previous reports describing NET-driven cytokine amplification in lupus monocytes.^37^ Notably, IFNα responses did not closely track with cGAS-STING activation, supporting the presence of additional interferon-inducing pathways in SLE monocytes. Together with our cytokine correlation analyses, these findings suggest that IFNα and IFNβ participate in partially distinct inflammatory networks, with IFNβ more closely associated with inflammatory mediators linked to monocyte activation.

The longitudinal design of our study enabled evaluation of disease-associated immune responses while minimising inter-individual variability. Nevertheless, circulating NET-associated markers varied considerably between patients and may reflect the dynamic balance between NET formation and clearance, which can also be influenced by immunosuppressive therapies.^38^^,^^39^ Importantly, reduced circulating NET abundance does not necessarily indicate reduced pathogenicity, as the immunogenicity of SLE-derived NETs is influenced by their molecular composition, including enrichment of oxidised mitochondrial DNA and autoantigens such as LL-37.^40^^,^^41^ Accordingly, the pathogenic potential of SLE-derived NETs may be driven by their composition, rather than their abundance.

While murine lupus models suggest that cGAS-STING activation does not exacerbate autoimmunity,^42^ our data are consistent with recent studies showing that NETs present in human SLE plasma effectively activate this pathway in primary human monocytes.^16^ These discrepancies may reflect species-specific differences in cGAS-STING regulation, NET composition, and the chronic inflammatory environment characteristic of human SLE, but may also be influenced by differences in experimental design, model systems, disease context, or other factors.

Several limitations of this study should be acknowledged. First, the relatively small cohort size and limited patient numbers within each clinical subgroup precluded adequately powered multivariable analyses to assess the independent effects of medication use, comorbidities, and disease characteristics on NET-associated immune activation. Accordingly, this study was designed primarily to provide mechanistic insight into the role of NET-associated DNA in cGAS-STING signalling rather than to establish clinical predictors of pathway activation. Second, although longitudinal sampling enabled evaluation of disease-associated changes within individual patients, differences in sample availability across disease activity states limited modelling of individual disease trajectories. In addition, one patient initiated anifrolumab treatment at the 12-month follow-up, which may have influenced interferon-related outcomes and should be considered when interpreting the longitudinal data. Finally, although SLE plasma activated the cGAS-STING pathway and induced type I IFN responses in monocytes, and *in-vitro*-generated NETs reproduced aspects of this response, the specific plasma component(s) mediating this response remain to be defined. The *in vivo* mechanisms governing NET DNA uptake, intracellular trafficking, and cell-specific pathway activation remain to be explored.

Our findings support a potential mechanistic link between NET-associated DNA and cGAS-STING-dependent type I IFN responses in human monocytes, providing a framework for understanding how NET-derived nucleic acids may contribute to persistent inflammation in SLE. Future studies in larger longitudinal cohorts representing diverse SLE phenotypes are needed to validate the relationship between NET-associated markers, cGAS-STING activation, and interferon responses. Such studies could evaluate the relationship of this pathway with longitudinal disease activity and prospective flare outcomes, and assess the influence of clinical variables on NET-mediated immune responses. Further mechanistic studies should define how NET-associated DNA engages cGAS-STING by investigating NET uptake, intracellular trafficking, and DNA-sensing pathways in monocytes. Also, precisely characterising the molecular composition of NET-associated DNA, including the contributions of nuclear DNA, mitochondrial DNA, and oxidatively modified DNA, together with identifying additional immunostimulatory NET components suggested by the persistent cytokine responses to DNA-depleted NET preparations, will further clarify the mechanisms of NET-mediated immune activation. Finally, NET-induced pSTING and pTBK1 activation signatures warrant evaluation in larger cohort to determine whether they have utility as biomarkers of disease activity, while their potential for flare prediction and patient stratification provides an important direction for future investigation.

## Contributors

Xiaolin Cao: Conceptualisation, Methodology, Investigation, Data curation, Formal analysis, Visualisation, Writing–original draft, Writing–review & editing.

Dagmar Duijzer: Methodology, Investigation, Data curation.

Tineke Kardol-Hoefnagel: Methodology, Investigation, Data curation, Writing–review & editing.

Bettina C. Geertsema-Hoeve: Resources, Investigation, Project administration, Writing–review & editing.

Jeanette H. W. Leusen: Supervision, Project administration.

Maarten Limper: Conceptualisation, Investigation, Resources, Project administration, Supervision, Visualisation, Writing–review & editing.

Marianne Boes: Conceptualisation, Investigation, Supervision, Project administration, Visualisation, Writing–original draft, Writing–review & editing.

Xiaolin Cao and Marianne Boes accessed and verified the underlying data reported in the manuscript. All authors read and approved the final version of the manuscript.

## Data sharing statement

The data supporting the findings of this study are available from the corresponding author upon reasonable request. The data are not publicly available because they contain information derived from human participants and may be subject to privacy and ethical restrictions. Requests for access will be considered by the corresponding author and may require appropriate ethical and data-protection approval.

## Declaration of interests

All authors declare no competing interests.
