# Supplementary material for: NET-derived DNA is associated with cGAS-STING and type I IFN responses, as supported by monocyte stimulation cultures using plasma from female patients with SLE
Source: eBioMedicine. 2026 Oct 7;133:106507. doi: 10.1016/j.ebiom.2026.106507 (PMC13675855; doi:10.1016/j.ebiom.2026.106507)
Supplement: Supplementary file 1 [file mmc1.pdf]

Supplementary Figure 1: Plasma interferon levels and their correlations with disease activity in SLE.

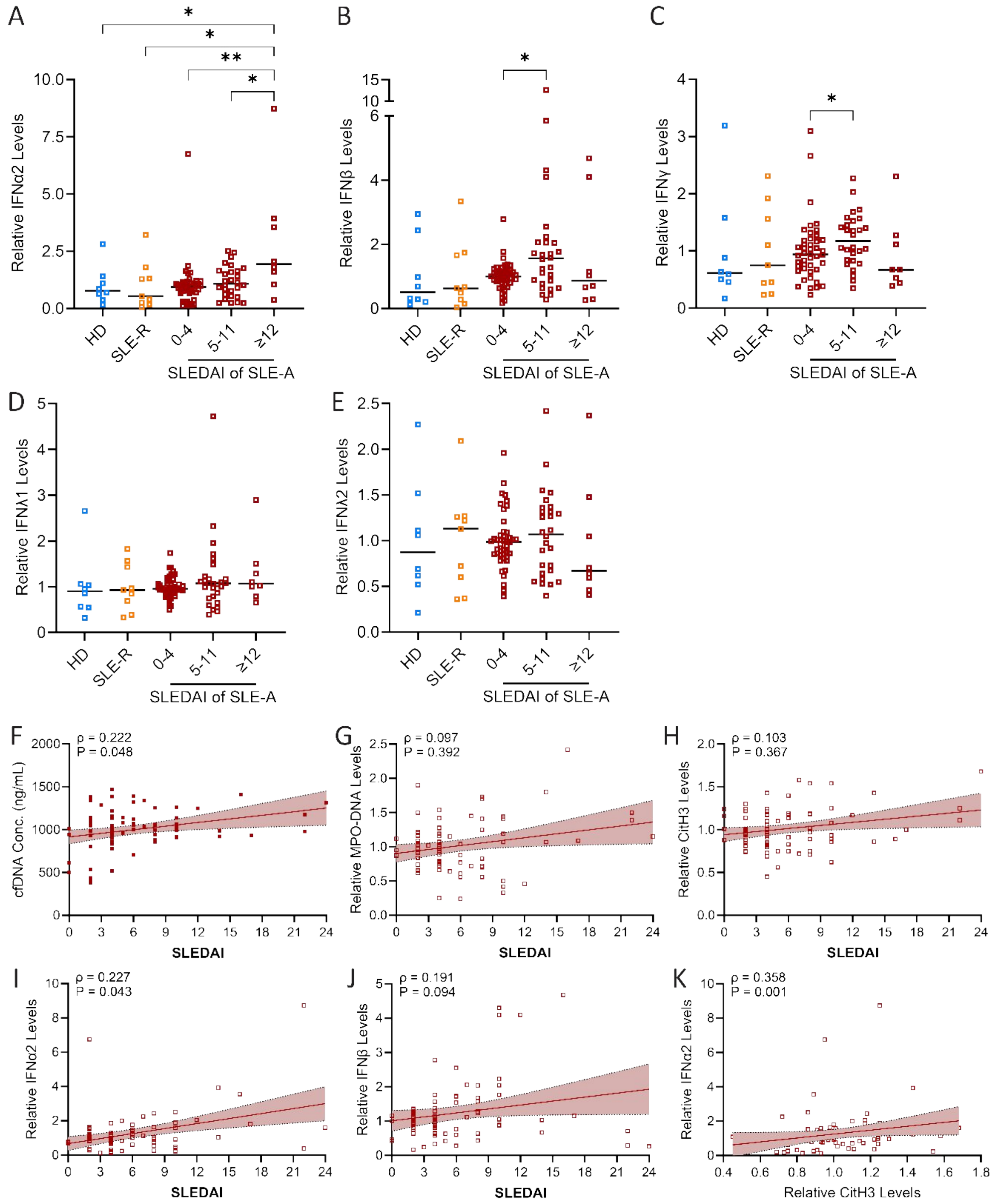

A. Relative IFNα2 levels in plasma samples from healthy donors (HD), patients with SLE in remission (SLE-R), and patients with active SLE (SLE-A) stratified according to disease activity measured by SLEDAI scores. B-E. Relative IFNβ, IFNγ, IFNλ1, and IFNλ2 levels in plasma samples from HD and patients with SLE groups. Bars represent median values. HD, n=8; SLE-R, n=9; SLEDAI 0-4, n=44; SLEDAI 5-11, n=28; SLEDAI ≥12, n=8. The Kruskal-Wallis test was used for multiple comparisons. *p<0.05; **p<0.01. F. Spearman correlation between plasma cfDNA concentrations and SLEDAI scores in active SLE samples (n=80). G. Spearman correlation between relative MPO-DNA levels and SLEDAI scores in active SLE

plasma samples (n=80). H. Spearman correlation between relative citrullinated histone H3 (CitH3) levels and SLEDAI scores in active SLE plasma samples (n=80). I. Spearman correlation between relative IFNα2 levels and SLEDAI scores in active SLE plasma samples (n=80). J. Spearman correlation between relative IFNβ levels and SLEDAI scores in active SLE plasma samples (n=80). K. Spearman correlation between relative IFNα2 levels and relative CitH3 levels in active SLE plasma samples (n=80).

Supplementary Figure 2: Effects of SLE plasma on monocyte subsets and surface marker expression.

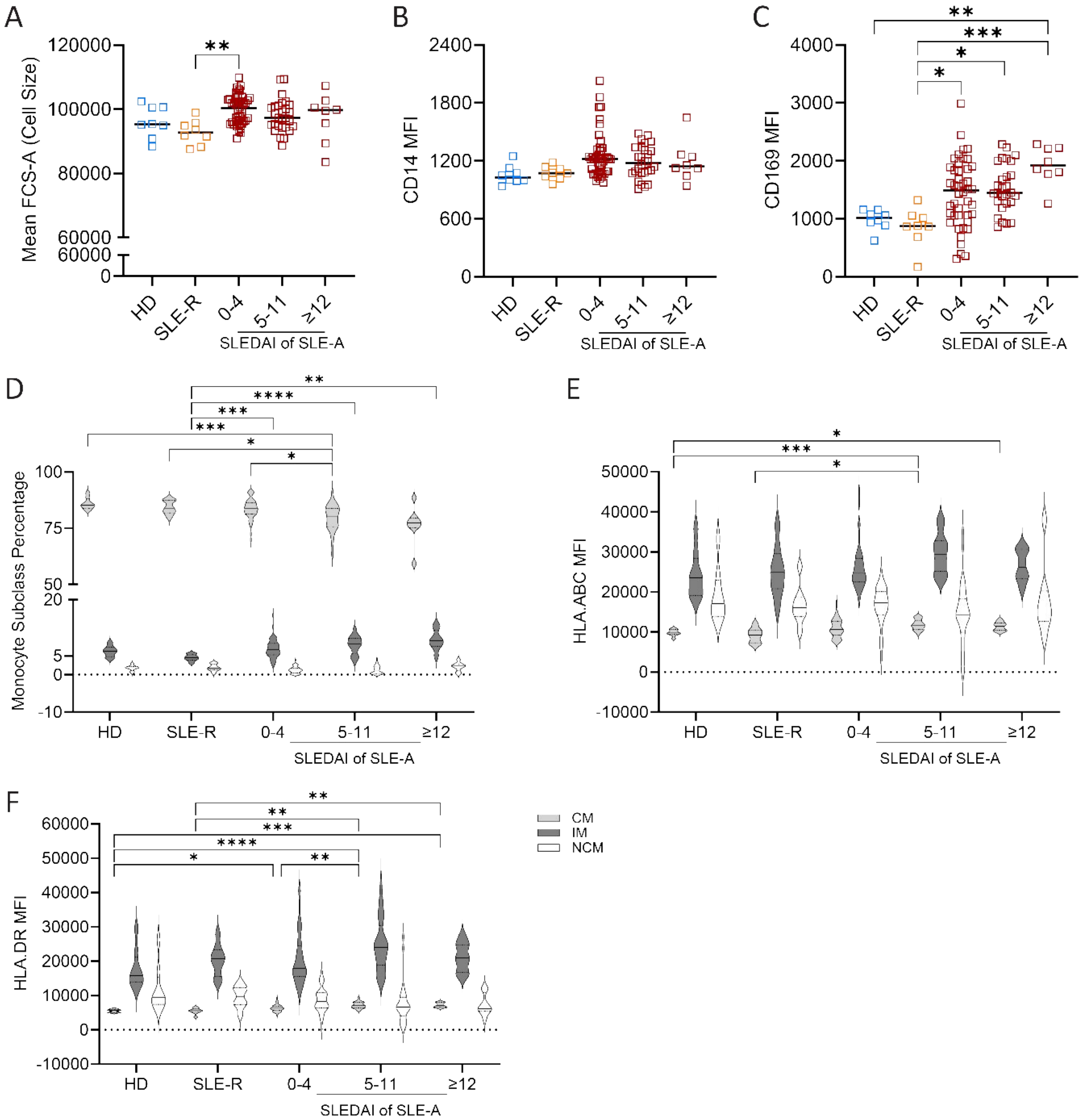

A. Cell size of monocytes from healthy donors following stimulation with control plasma or plasma from patients with SLE from the indicated groups (HD, SLE-R, and three active SLE groups stratified by SLEDAI scores). B. MFI of CD14 on monocytes following plasma stimulation. C. MFI of CD169 on monocytes following plasma stimulation. A-C: Bars represent median values. D. Percentage of monocyte subsets, including classical monocytes

(CM), intermediate monocytes (IM), and non-classical monocytes (NCM), following plasma stimulation. E. MFI of HLA-ABC in monocyte subsets (CM, IM, and NCM) following plasma stimulation. F. MFI of HLA-DR in monocyte subsets (CM, IM, and NCM) following plasma stimulation. D-F: Median values (solid line) and quartiles (dotted lines) are indicated. MFI represents Mean fluorescence intensity. HD, n=8; SLE-R, n=8; SLEDAI 0-4, n=44; SLEDAI 5-11, n=28; SLEDAI ≥12, n=8. The Kruskal-Wallis test was used for multiple comparisons. *p<0.05; **p<0.01; ***p<0.001; ****P < 0.0001.

Supplementary Figure 3 Association of monocyte cGAS-STING pathway activation with disease activity and IFNβ production.

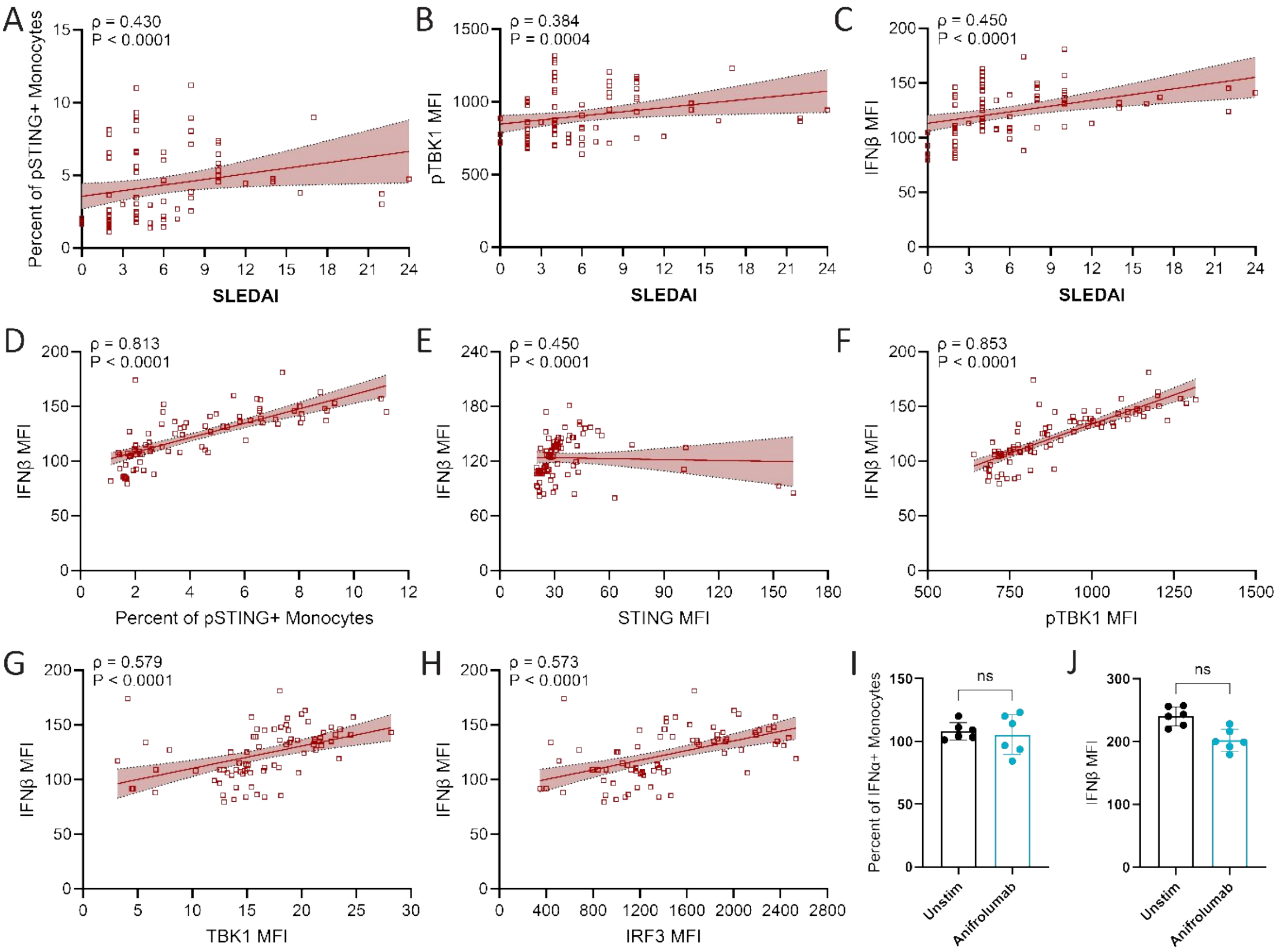

A. Spearman correlation between the percentage of STING-positive monocytes and plasma SLEDAI scores. B. Spearman correlation between pTBK1 MFI and plasma SLEDAI scores. C. Spearman correlation between IFNβ MFI in monocytes and plasma SLEDAI scores. D. Spearman correlation between IFNβ MFI in monocytes and the percentage of pSTING-positive monocytes. E. Spearman correlation between IFNβ MFI and STING MFI in monocytes. F. Spearman correlation between IFNβ MFI and pTBK1 MFI in monocytes. G. Spearman correlation between IFNβ MFI and TBK1 MFI in monocytes. H. Spearman correlation between IFNβ MFI and IRF3 MFI in monocytes. A-H: n=80 I. Percentage of IFNα-positive monocytes following stimulation in the absence or presence of anifrolumab (n=6). J. MFI of IFNβ in monocytes following stimulation in the absence or presence of anifrolumab (n=6). I-J: The Wilcoxon matched-pairs signed-rank test was used for paired comparisons. ns, p≥0.05. MFI represents Mean fluorescence intensity.

Supplementary Figure 4 Effects of NETs and DeNETs on STING, TBK1, and IRF3 expression and type II and type III IFN production by monocytes.

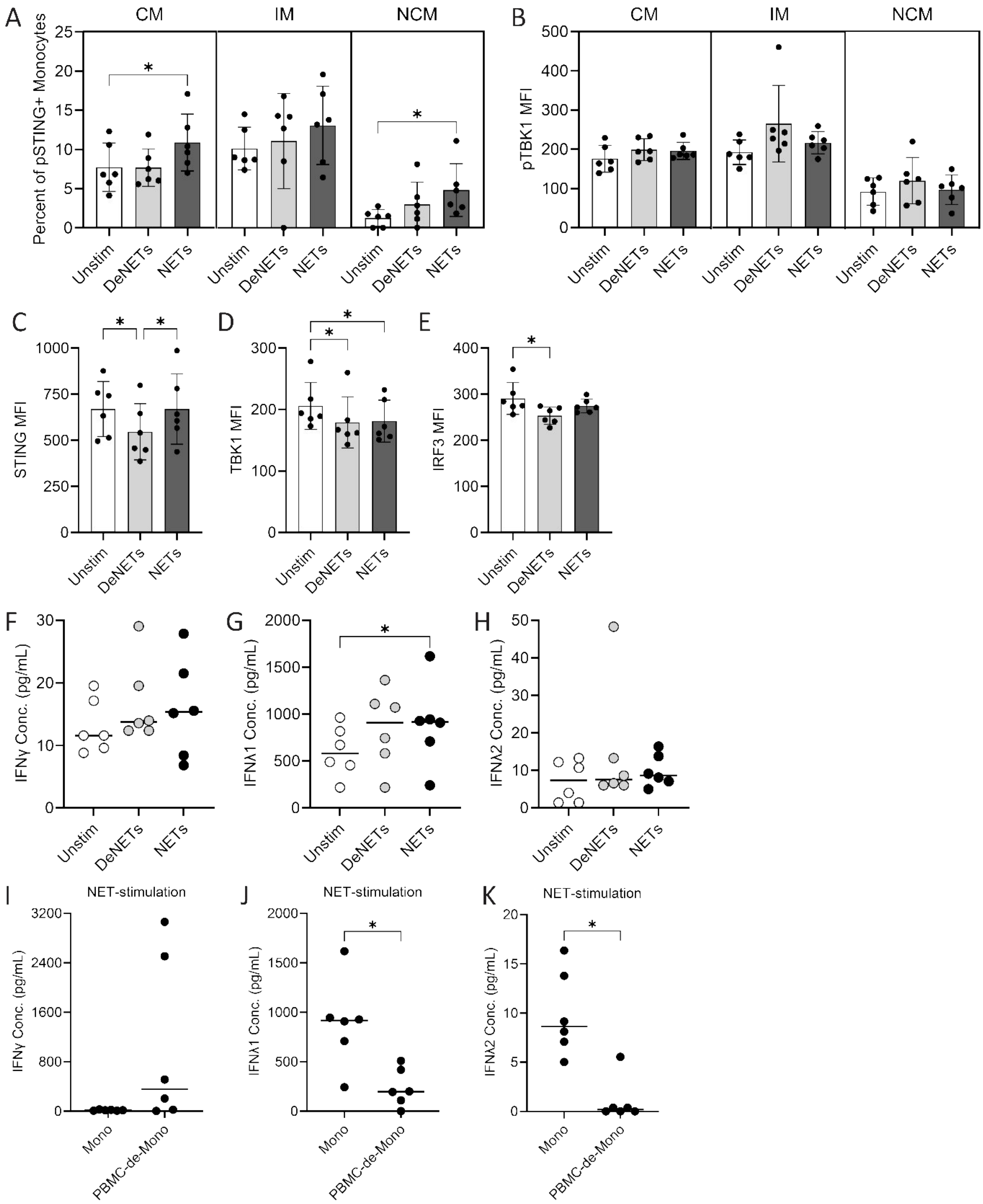

A. Percentage of pSTING-positive monocyte subclasses (CM, IM, NCM) after 24-hour stimulation. B. MFI of pTBK1 of monocyte subclasses (CM, IM, NCM) after 24-hour stimulation. C. MFI of STING in monocytes following 24-hour stimulation. D. MFI of TBK1 in monocytes following 24-hour stimulation. E. MFI of IRF3 in monocytes following 24-hour stimulation. A-E: Bars represent mean values, with error bars indicating standard deviation. F. IFNγ concentration in monocyte supernatants following stimulation. G. IFNλ1 concentration in monocyte supernatants following stimulation. H. IFNλ2 concentration in monocyte

supernatants following stimulation. I. IFNγ secretion from monocytes compared with the same number of PBMCs depleted of monocytes from the same donors. J. IFNλ1 secretion from monocytes compared with the same number of PBMCs depleted of monocytes from the same donors. K. IFNλ2 secretion from monocytes compared with the same number of PBMCs depleted of monocytes from the same donors. F-K: Bars represent median values. MFI, mean fluorescence intensity. n=6. The Friedman test with Dunn's multiple comparisons test was used for multiple comparisons. *$p<0.05$.

Supplementary Figure 5 Associations between plasma IFNβ and inflammatory mediators in active SLE.

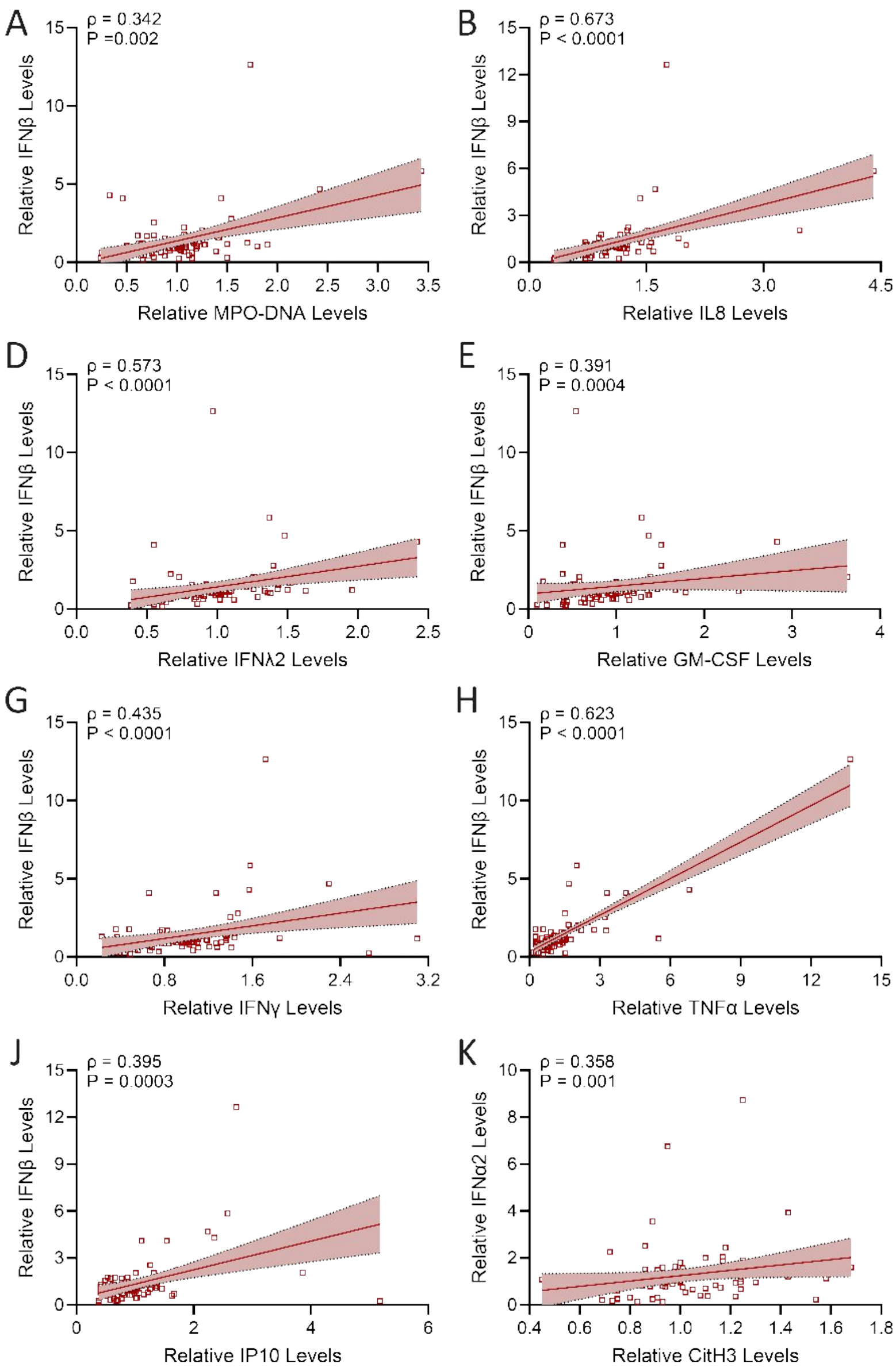

Spearman correlation between plasma IFNβ levels and plasma levels of MPO-DNA (A), IL-8 (B), IFNλ1 (C), IFNλ2 (D), GM-CSF (E), IL-1β (F), IFNγ (G), TNFα (H), IL-12p70 (I), and IP-10 (J) in active SLE samples (n=80). (K) Spearman correlation between plasma IFNα2 and CitH3 levels in active SLE samples (n=80). Spearman's ρ and p values are indicated on the plots.

Supplementary Table 1
Age and Sex Information of Plasma Donors

MDS	Age	Sex	SLE-R	Age	Sex	Comorbidities	Medication	Ethnicity	SLE-A	Age	Sex	Ethnicity	Comorbidities
HD1	36	Female	SLE1	39	Female	None	HCQ, AZA	White	SLE10	40	Female	White	Sarcoidosis, ventricular septal defect
HD2	42	Female	SLE2	43	Female	Type 2 diabetes mellitus	HCQ, MMF	White	SLE11	41	Female	White	None
HD3*	66	Female	SLE3*	36	Female	None	MMF	White	SLE12	62	Female	White	Sjogrens' disease, aortic valve insufficiency
HD4	51	Female	SLE4	53	Female	Spleen excision	HCQ	White	SLE13	51	Female	White	None
HD5	54	Female	SLE5	58	Female	Graves' disease	HCQ	White	SLE14	58	Female	White	Sjogrens' disease
HD6	35	Female	SLE6	35	Female	IBS	HCQ	White	SLE15	32	Female	White	None
HD7	54	Female	SLE7	54	Female	None	None	White	SLE16	54	Female	White	None
HD8	66	Female	SLE8	64	Female	Recurrent psychosis, Parkinson's disease, breast cancer	HCQ	Black	SLE17	62	Female	White	Spleen excision
HD9	50	Female	SLE9	47	Female	None	HCQ, MTX	White	SLE18	52	Female	Asian	Osteoporotic vertebral fracture

Note: *In experiments indicated n=8, donors were absent. HCQ, hydroxychloroquine; AZA, azathioprine; MMF, mycophenolate mofetil; MTX, methotrexate.

Supplementary Table 2
Medical Record of Patients with Active SLE

SLE-A	Timepoint of flare	SLEDAI at timepoint of flare	Treament_at_flare	Treatment changed due to flare?
SLE10	T12	17	HCQ 200 mg twice daily	Yes, anifrolumab started
SLE11	T24	6	HCQ 200 mg twice daily, prednisone 10 mg/day, tacrolimus 15 mg/day	Tacrolimus discontinued, prednisone increased to 20 mg/day, anifrolumab started
SLE12	T18	18	HCQ 200 mg twice daily, AZA 100 mg/day, prednisone 5 mg/day	Yes, prednisone increased to 7.5 mg/day
SLE13	T6	6	Prednisone 5 mg/day, HCQ 200 mg twice daily, AZA 150 mg/day	No

SLE14	T3	10	Prednisone 2.5 mg/day, HCQ 200 mg once or twice daily, ciclosporine 75 mg twice daily, belimumab 200 mg/week	Yes, prednisone increased to 5 mg/day
SLE15	Between T18 & T21	T18: 2<br>T21: 0	HCQ 200 mg/day	No
SLE16	Between T9 & T12	T9: 8<br>T12: 10	Belimumab, MTX 20 mg, prednisone 20 mg/day, HCQ	No
SLE17	Between T21 & T24	T21: 3<br>T24: 2	Belimumab	Yes, prednisone started
SLE18	Between T9 & T12	T9: 16<br>T12: 4	MTX	Yes, MTX discontinued, rituximab started, methylprednisolone administered twice

Note: T3=3 month… T24=24 month. HCQ, hydroxychloroquine; AZA, azathioprine; MTX, methotrexate.

Supplementary Table 3
Antibodies Used for Flow Cytometry

Target antigen	Fluorochrome	Cat. No.	Supplier	Research Resource Identifier (RRID)
pSTING (S366)	Alexa® 488	41622S	Cell Signalling Technology	AB_2799204
IFNβ	FITC	BMS1044FI	Invitrogen	AB_11220279
IRF3	APC	IC4019A	R&D systems	AB_3655687
Viability Dye	eFluo780	65-0865-18	eBioscience	Not applicable
pTBK1 (pS172)	Alexa Fluor ® 647	558603	BD Biosciences	AB_647128
STING	PE	564965	BD Biosciences	AB_2739026
TBK1	PE	558696	BD Biosciences	AB_1645537
HLA-ABC	PE-Cy7	561349	BD Biosciences	AB_10612559
IFNα[2b]	V450	561382	BD Biosciences	AB_10716058
HLA-DR	Brilliant Violet 421	307636	Biolegend	AB_2561831
CD14	V500	561391	BD Biosciences	AB_10611856
CD16	Brilliant Violet 785	302046	Biolegend	AB_2563803

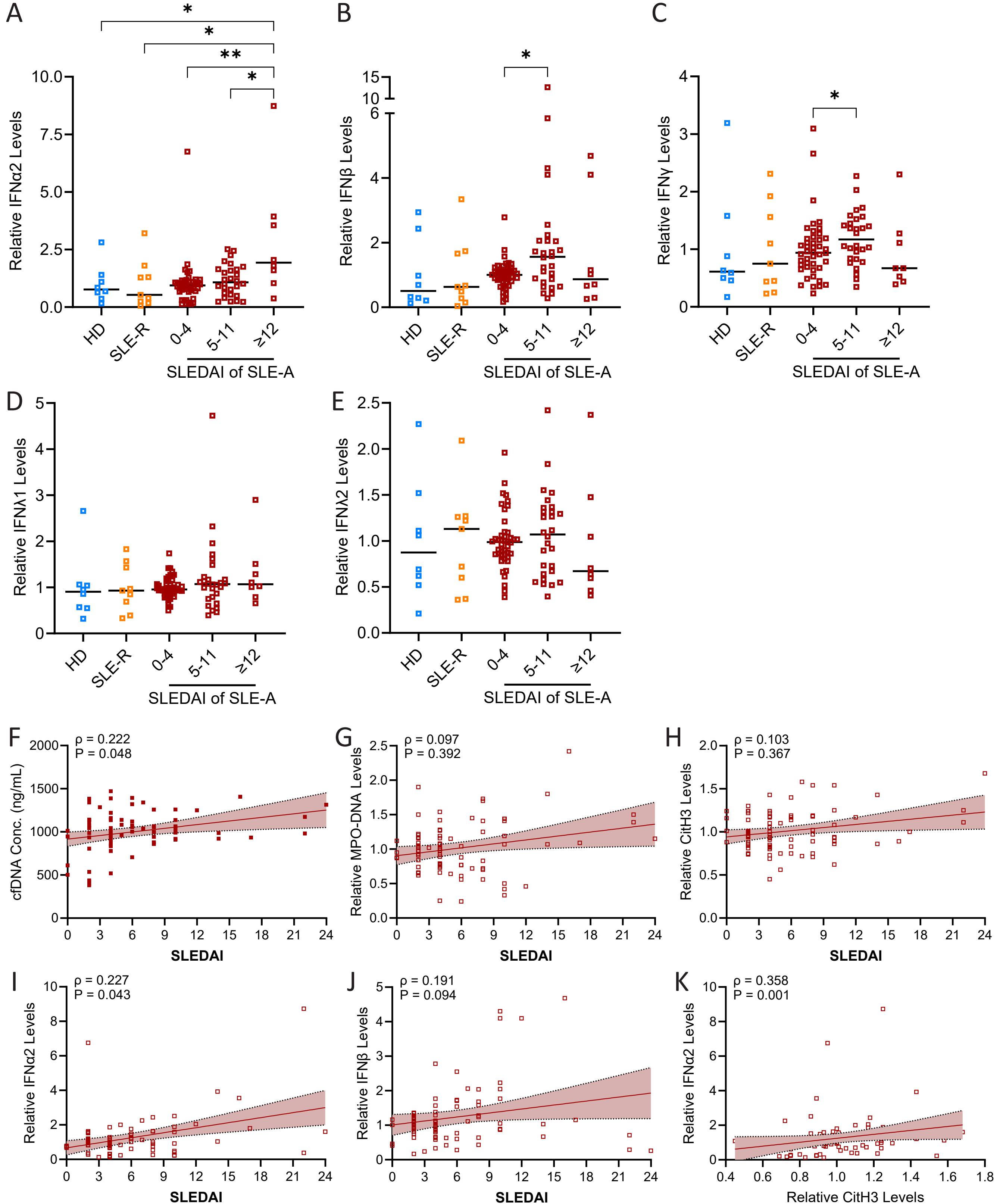
A
Relative IFNα2 Levels
HD
SLE-R
0-4
5-11
≥12
SLEDAI of SLE-A
B
Relative IFNβ Levels
C
Relative IFNγ Levels
D
Relative IFNλ1 Levels
E
Relative IFNλ2 Levels
F
ρ = 0.222
P = 0.048
cfDNA Conc. (ng/mL)
SLEDAI
G
ρ = 0.097
P = 0.392
Relative MPO-DNA Levels
H
ρ = 0.103
P = 0.367
Relative CitH3 Levels
I
ρ = 0.227
P = 0.043
J
ρ = 0.191
P = 0.094
K
ρ = 0.358
P = 0.001
Relative CitH3 Levels

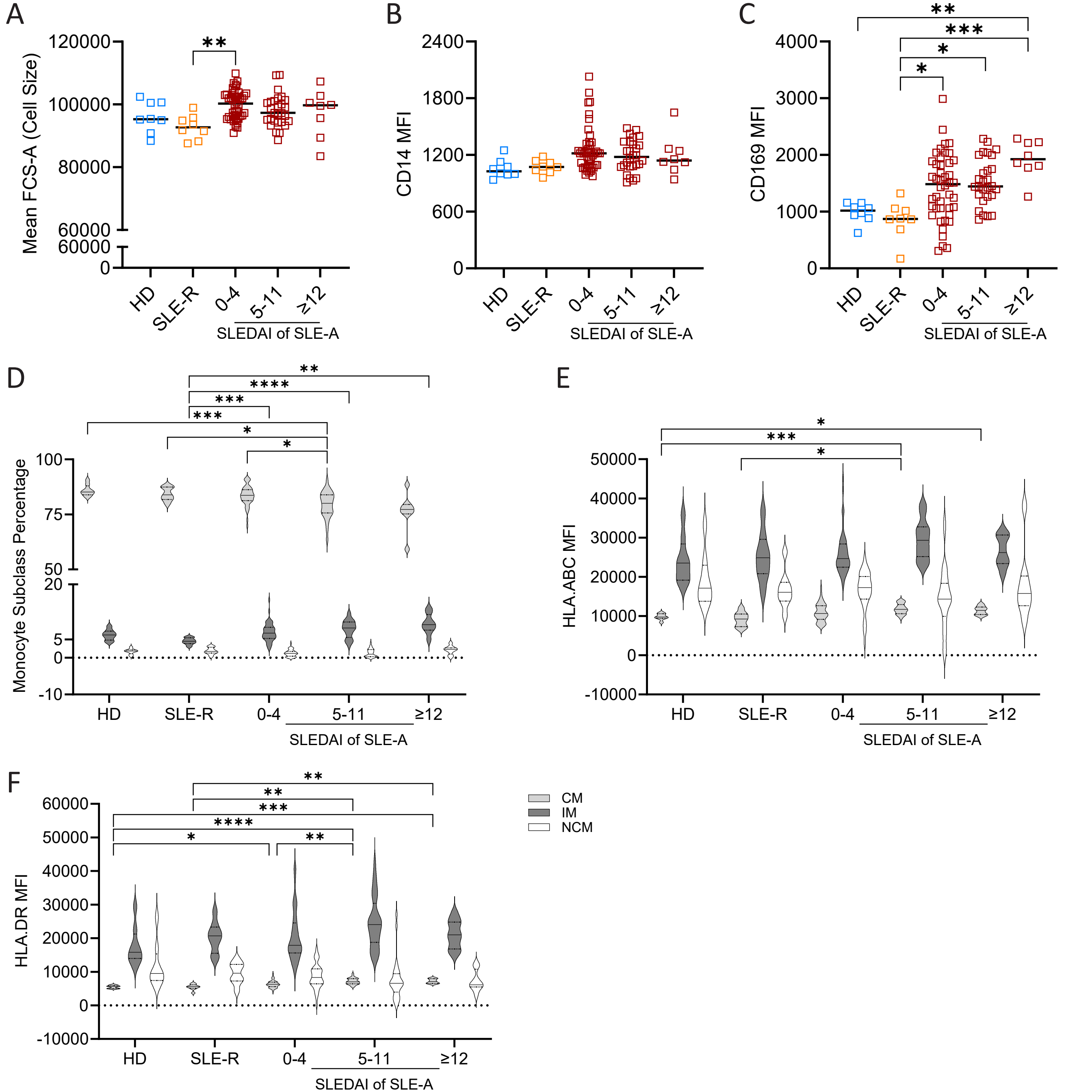
A
B
C
D
E
F
Mean FCS-A (Cell Size)
CD14 MFI
CD169 MFI
Monocyte Subclass Percentage
HLA.ABC MFI
HLA.DR MFI
HD
SLE-R
0-4
5-11
≥12
SLEDAI of SLE-A
CM
IM
NCM

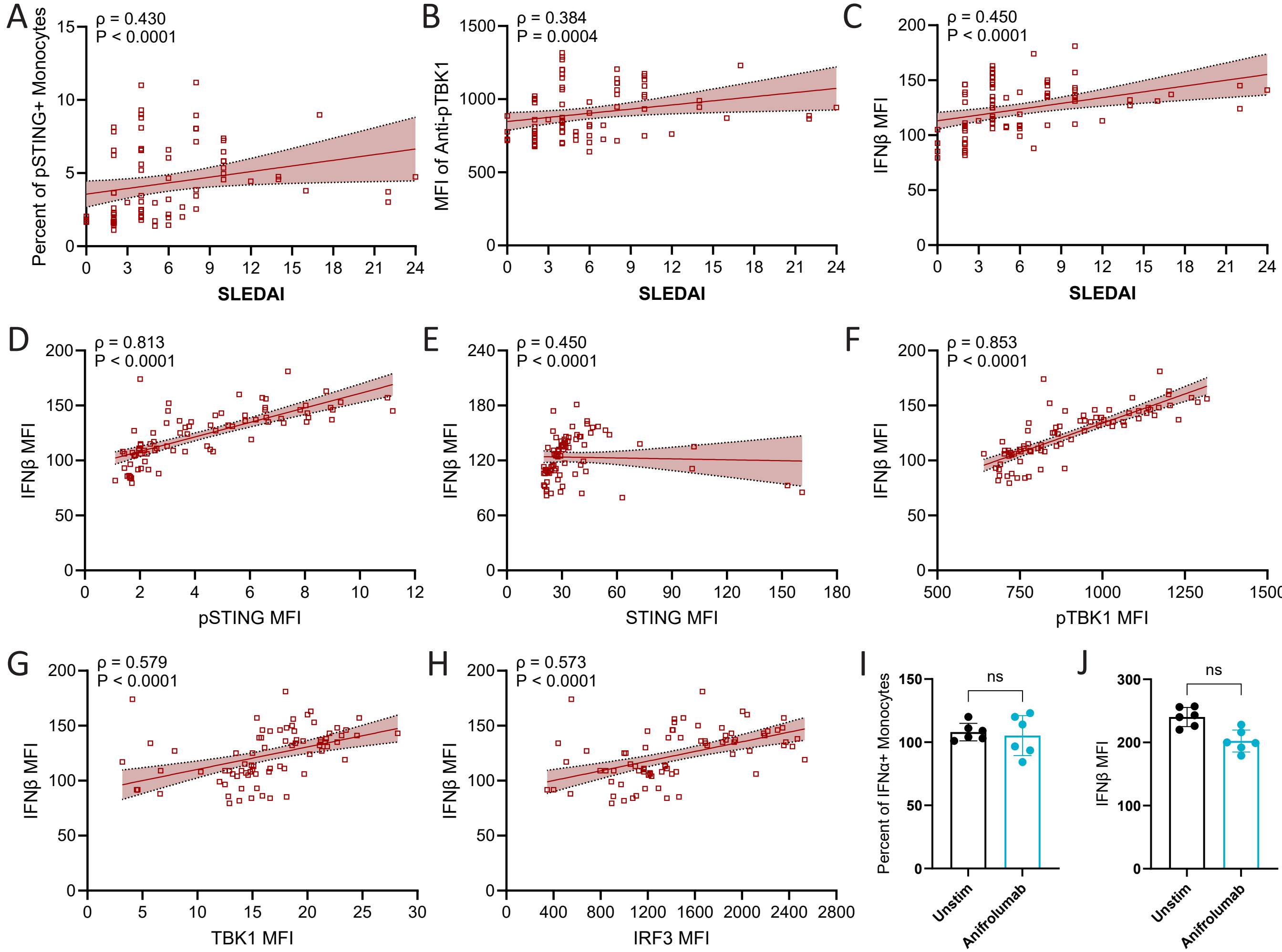

A
ρ = 0.430
P < 0.0001
Percent of pSTING+ Monocytes
SLEDAI
B
ρ = 0.384
P = 0.0004
MFI of Anti-pTBK1
SLEDAI
C
ρ = 0.450
P < 0.0001
IFNβ MFI
SLEDAI
D
ρ = 0.813
P < 0.0001
IFNβ MFI
pSTING MFI
E
ρ = 0.450
P < 0.0001
IFNβ MFI
STING MFI
F
ρ = 0.853
P < 0.0001
IFNβ MFI
pTBK1 MFI
G
ρ = 0.579
P < 0.0001
IFNβ MFI
TBK1 MFI
H
ρ = 0.573
P < 0.0001
IFNβ MFI
IRF3 MFI
I
ns
Percent of IFNα+ Monocytes
Unstim
Anifrolumab
J
ns
IFNβ MFI
Unstim
Anifrolumab

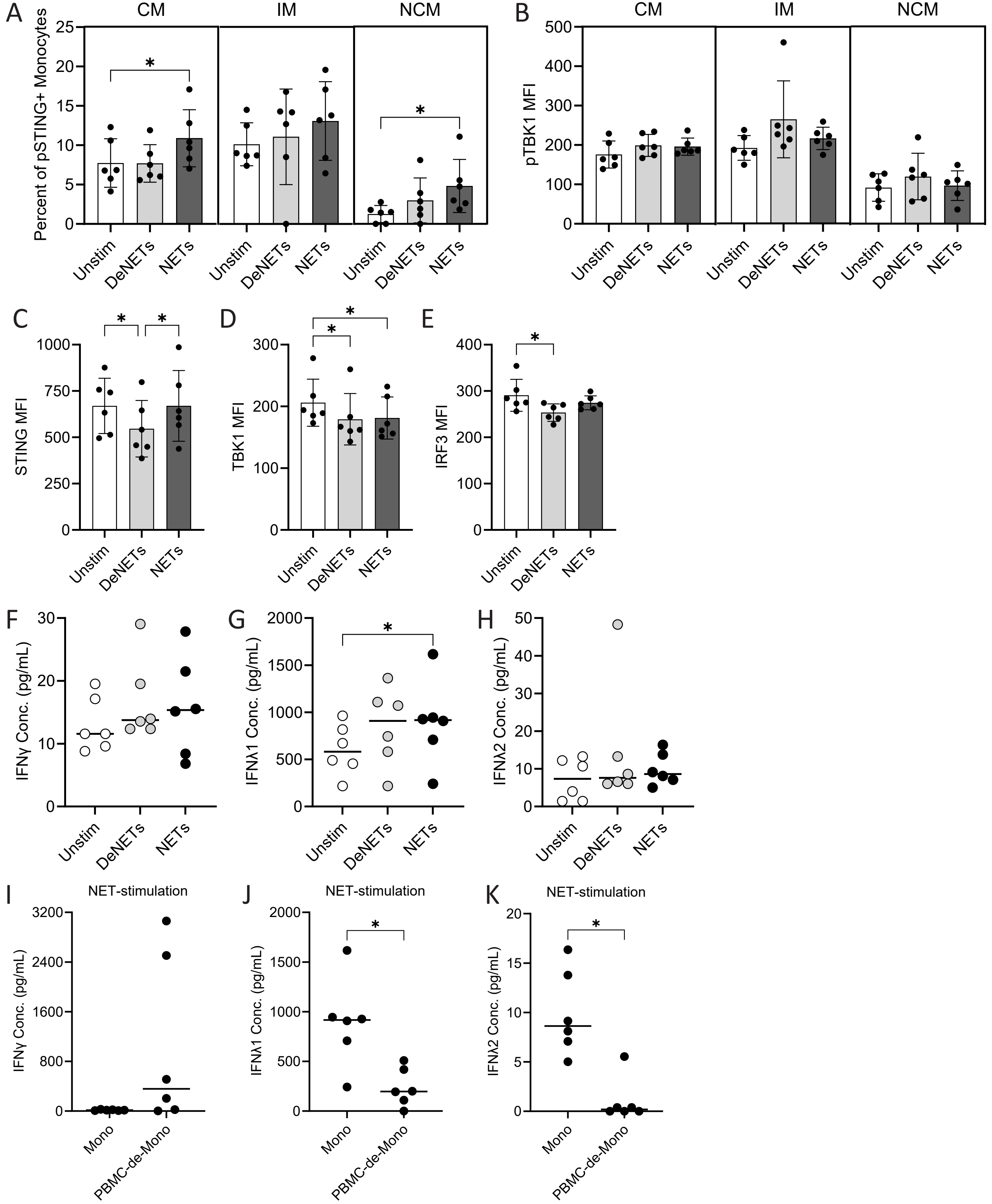

A
CM
IM
NCM
Percent of pSTING+ Monocytes
Unstim
DeNETs
NETs
*
B
pTBK1 MFI
C
STING MFI
D
TBK1 MFI
E
IRF3 MFI
F
IFNγ Conc. (pg/mL)
G
IFNλ1 Conc. (pg/mL)
H
IFNλ2 Conc. (pg/mL)
I
NET-stimulation
J
K
Mono
PBMC-de-Mono

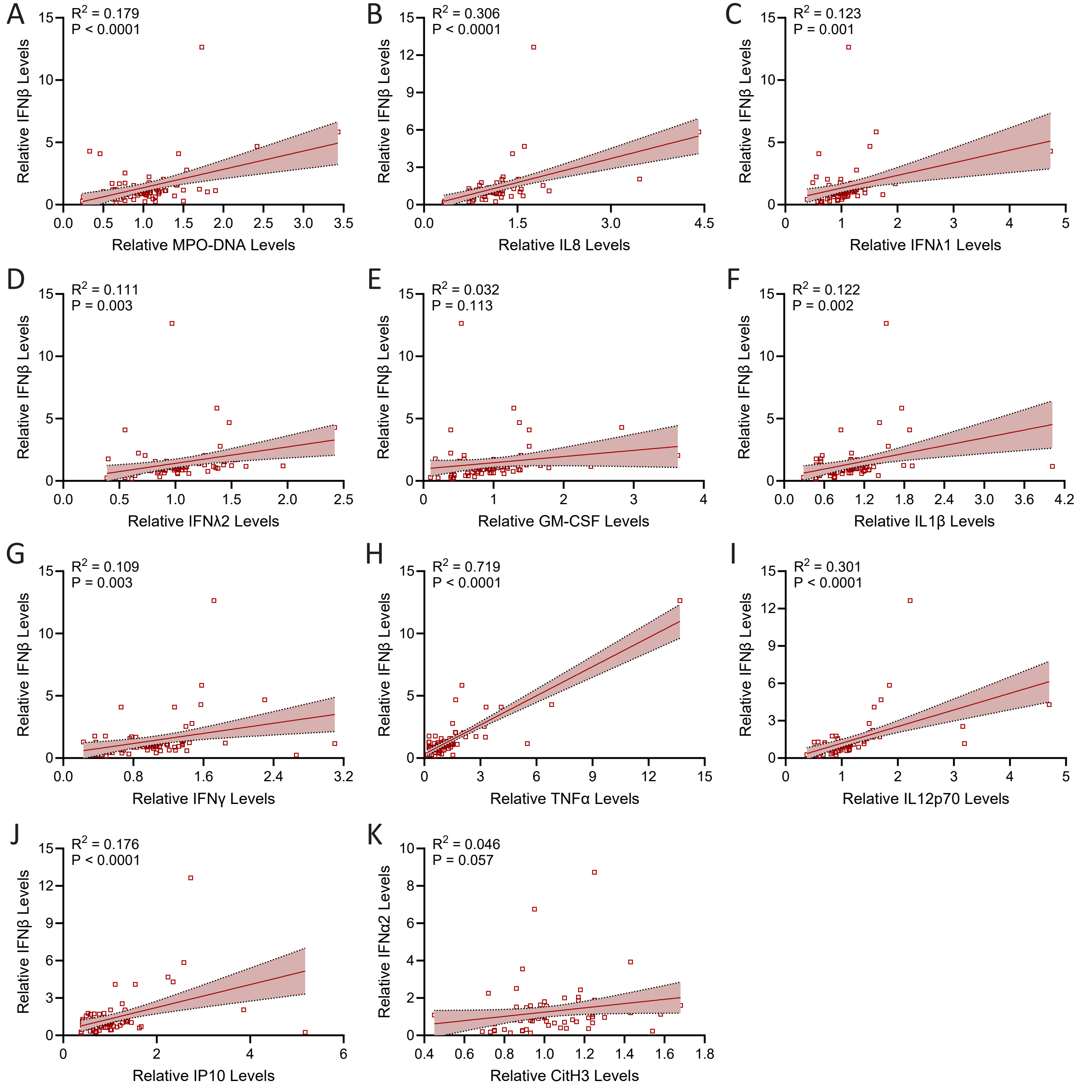
A
R^2 = 0.179
P < 0.0001
Relative IFNβ Levels
Relative MPO-DNA Levels
B
R^2 = 0.306
P < 0.0001
Relative IFNβ Levels
Relative IL8 Levels
C
R^2 = 0.123
P = 0.001
Relative IFNβ Levels
Relative IFNλ1 Levels
D
R^2 = 0.111
P = 0.003
Relative IFNβ Levels
Relative IFNλ2 Levels
E
R^2 = 0.032
P = 0.113
Relative IFNβ Levels
Relative GM-CSF Levels
F
R^2 = 0.122
P = 0.002
Relative IFNβ Levels
Relative IL1β Levels
G
R^2 = 0.109
P = 0.003
Relative IFNβ Levels
Relative IFNγ Levels
H
R^2 = 0.719
P < 0.0001
Relative IFNβ Levels
Relative TNFα Levels
I
R^2 = 0.301
P < 0.0001
Relative IFNβ Levels
Relative IL12p70 Levels
J
R^2 = 0.176
P < 0.0001
Relative IFNβ Levels
Relative IP10 Levels
K
R^2 = 0.046
P = 0.057
Relative IFNα2 Levels
Relative CitH3 Levels
